# Algorithmic handwriting analysis of the Samaria inscriptions illuminates bureaucratic apparatus in biblical Israel

**DOI:** 10.1371/journal.pone.0227452

**Published:** 2020-01-22

**Authors:** Shira Faigenbaum-Golovin, Arie Shaus, Barak Sober, Eli Turkel, Eli Piasetzky, Israel Finkelstein

**Affiliations:** 1 Department of Applied Mathematics, Tel Aviv University, Tel Aviv, Israel; 2 Jacob M. Alkow Department of Archaeology and Ancient Near Eastern Civilizations, Tel Aviv University, Tel Aviv, Israel; 3 Department of Genetics, Harvard Medical School, Boston, MA, United States of America; 4 Department of Mathematics, Duke University, Durham, NC, United States of America; 5 School of Physics and Astronomy, Tel Aviv University, Tel Aviv, Israel; University of Iowa, UNITED STATES

## Abstract

Past excavations in Samaria, capital of biblical Israel, yielded a corpus of Hebrew ink on clay inscriptions (ostraca) that documents wine and oil shipments to the palace from surrounding localities. Many questions regarding these early 8^th^ century BCE texts, in particular the location of their composition, have been debated. Authorship in countryside villages or estates would attest to widespread literacy in a relatively early phase of ancient Israel's history. Here we report an algorithmic investigation of 31 of the inscriptions. Our study establishes that they were most likely written by two scribes who recorded the shipments in Samaria. We achieved our results through a method comprised of image processing and newly developed statistical learning techniques. These outcomes contrast with our previous results, which indicated widespread literacy in the kingdom of Judah a century and half to two centuries later, ca. 600 BCE.

## Introduction

The question of literacy in ancient (biblical) Israel is crucial for biblical exegesis and related fields. In a recent article [1] we dealt with the corpus of ostraca (ink inscriptions on clay sherds) unearthed at the desert fortress of Arad in Judah (the southern of the two Hebrew kingdoms), dated to ca. 600 BCE. We introduced an algorithmic framework that allowed us to estimate that the 18 inscriptions investigated were authored by at least four individuals (six if one adds information provided by the texts), representing different positions in the hierarchy of Judah's military system. This indicates significant dissemination of literacy in Judah in the years before its destruction by Babylonia. In the current study we turn to a corpus of ostraca written 150–200 years earlier, uncovered in Samaria, the capital of Israel (the Northern Kingdom) [2], the stronger and more prosperous of the two Hebrew kingdoms, which competed with Damascus for domination of the Levant in the 9^th^ and 8^th^ centuries BCE.

According to the biblical account (1 Kings 16:24), as well as the reference to Israel in Assyrian sources, King Omri established Samaria as the capital of the kingdom in the early 9th century BCE and founded its first strong dynasty, the “House of Omri.” A second period of prosperity in Israel took place in the first half of the 8^th^ century. Excavations at the site, conducted at the beginning of the 20^th^ century [3], revealed a monumental and rich metropolis that lasted until the Assyrian takeover of the kingdom in 722/720 BCE.

**Fig 1:**
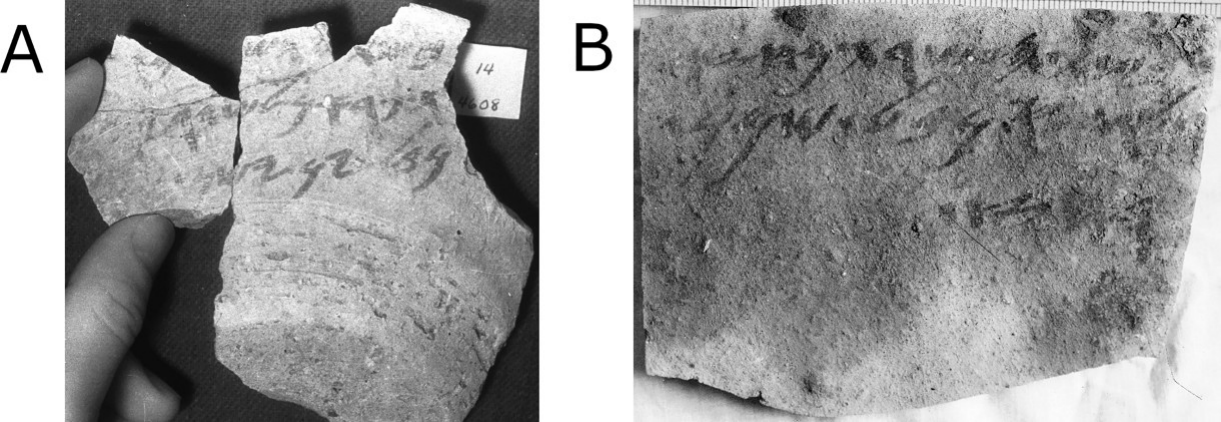
Examples of Samaria ostraca. (**A**) No. 14: “In the year ni[ne] from Az[…]t Par'an to Shemaryau jar of aged wine”; (**B**) No. 18: “In the year ten from Hazeroth to Gaddiyau jar of bath oil”.

**Fig 2:**
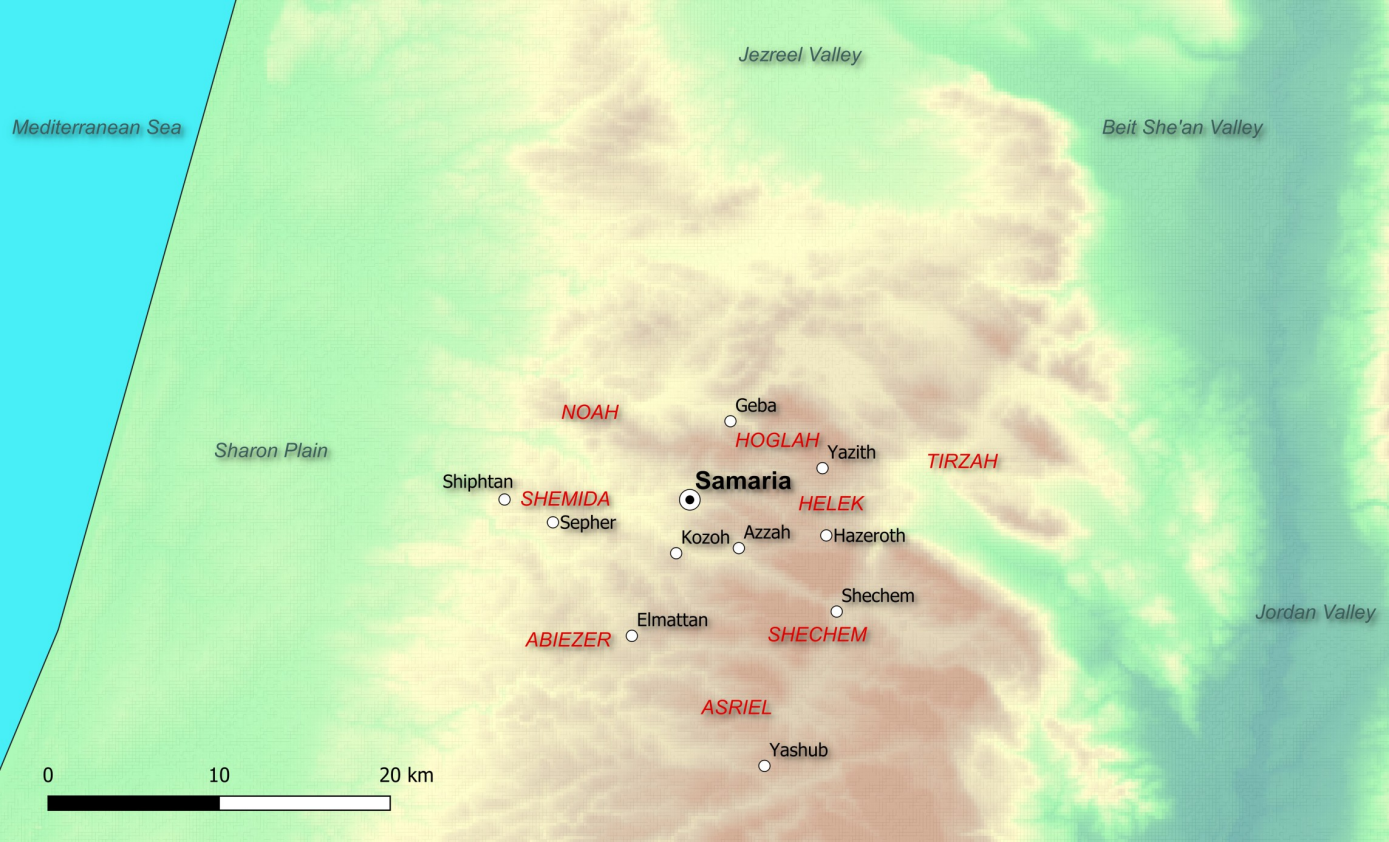
Map marking places and clans mentioned in the Samaria ostraca; based on [5,10]. In capital red are the names of the Manasseh tribal clans; in black are the names of identifiable localities.

The Samaria excavations yielded one of the richest corpora of Iron Age Hebrew inscriptions [3–5]. Based on paleographic considerations combined with information on regnal years in the texts (more below), this assemblage, comprised of ca. 100 short administrative texts, mainly ostraca (Fig 1), most probably dates to the first half of the 8^th^ century BCE [5–8]. The ostraca were found in a fill laid in preparation for the construction of a large building (see [8]; different view in [9]), labeled by the excavators [3] as the “Ostraca House.” The inscriptions record the delivery of wine and oil from villages or royal estates in the countryside to the capital. The telegraphic texts contain details such as regnal year of a king (citing the years 9, 10 and 15; see [6–8], toponym, name of clan (which the Bible lists in the genealogy of the tribe of Manasseh–e.g., Joshua 17:2–3), commodity type (wine/oil), and personal name, probably the sender or the recipient (for details see Table D in S1 File, example on Fig 1, and map on Fig 2). Due to its size and provenance, the Samaria corpus is crucial for reconstructing the history of the Northern Kingdom and biblical research, as well as the study of ancient Hebrew language and script.

Despite many years of extensive research, several issues related to the Samaria corpus are still debated (e.g., [11–14], summary in [9]). In particular, it is not clear whether the ostraca were composed at various sites in the highlands around the capital and dispatched to Samaria along with the provisions mentioned in them, or whether they were written in the capital, possibly when the shipments arrived. The former option would indicate dissemination of writing, at least in the administrative echelon of the kingdom of Israel, while the latter would provide evidence for the royal bureaucracy in the capital. Related issues are the number of individuals who authored the inscriptions (theoretically, there could have been itinerant scribes who traveled between royal estates), and the meaning of the information in the texts (the regnal year of the king, identification of the toponym, clan system, and function of the individuals mentioned; see Table D in S1 File). Interrelation between writers and these specific categories of information may indicate specialization within the scribes' milieus.

In a previous article ([1], strengthened by results in [15,16]), which dealt with the corpus of ostraca from the fortress of Arad in the desert fringe of southern Judah, we introduced an algorithmic framework capable of detecting statistically significant “separations” of authors within pairs of inscriptions in the assemblage. Our former techniques allowed us to estimate *the minimal* number of writers within the investigated corpus. As will be seen below, applying our method to the Samaria ostraca yields a small number of separations, and thus a small estimate of the minimal number of scribes. In fact, theoretically, *all* the separations could have been obtained by chance, representing “false positives,” despite an underlying single author. In order to deal with this possibility, answering the research questions presented in the current paper necessitated a revision and enhancement of our algorithmic apparatus. The main goal of the present research is to deduce *the most likely* number of scribes in the Samaria corpus, taking into account the possibilities for both “false positive” and “false negative” writers’ separations. The most likely estimate is sufficient for shedding light on some of the fundamental issues under discussion. To the best of our knowledge, this is the first attempt to estimate the most likely number of writers at Samaria, or any other ancient corpus, via classical paleographic or computational means.

## Materials and methods

### Datasets

This research was conducted on two datasets of ancient written material. The main assemblage was a corpus of 39 Hebrew ostraca found in Samaria, probably dating to the early 8^th^ century BCE. The study was performed on character reconstructions based on grayscale digital images of these inscriptions, scanned from negatives acquired by the Harvard expedition to Samaria [3] when they were unearthed at the beginning of the 20^th^ century. All reconstructions, performed by methods specified in the S1 File, Section 1, are available in [17].

Permission for research and publication of the results based on the negatives were obtained from the Harvard Semitic Museum. We refer to Texts # 2, 5, 6, 7, 8, 11, 12, 14, 15, 16a, 17a, 17b, 18, 19, 20, 21, 22, 24a, 29, 33, 34, 35, 36, 38, 40, 42, 43, 44, 45, 51, 52, 53, 54, 55, 56, 57, 59, 61 and 62, as well as the relatively short ostraca 11, 15, 17b, 33, 34, 40, 44 and 61, which were used for statistical enrichment of the algorithm. The registration numbers, and other details regarding the inscriptions, are provided in the S1 File, Section 1. All necessary permits were obtained for the described study, which complied with all relevant regulations.

A second dataset, utilized for the confusion matrices estimation (see below), contained information from a corpus of 16 Hebrew ostraca found at the Arad fortress which date to ca. 600 BCE. This dataset was published in [13].

### Algorithmic apparatus

Several factors can hamper an algorithmic analysis of ancient Hebrew ostraca via readily available means. First, the poor state of preservation of the ostraca (Fig 1) cannot be fully remedied by existing image acquisition methods [18–24]. Second, the imperfect digital images present a challenge for image segmentation and enhancement methods [25,26]. Third, although the task of identifying writers in handwritten texts has been addressed in previous literature (e.g., [27–32]), researchers presuppose a reference dataset with annotated authorship be used for training purposes, which is not present in our case. Additionally, the above publications do not aim directly at recognizing or distinguishing authors. Instead, they focus on finding a distance between inscriptions, which is useful only in limited scenarios (e.g., finding the k-most similar texts to a given one). In other words, the estimation of the number of writers in a given corpus has received little attention. In our previous publication [1], an algorithm yielding a *lower bound* for the number of authors in a group of inscriptions was introduced. Here we aim to provide the *maximum likelihood estimate* (MLE) for the number of hands in a corpus.

The algorithmic framework of this article consists of two consecutive stages. The goal of the first stage, presented in [1], is to establish separations between authors of every pair of inscriptions within the corpus. An improved version of this algorithm has been applied to the Samaria corpus. The purpose of the second, newly developed stage is to establish the most likely number of scribes within a given corpus of documents. The general idea is to obtain a number of *hands’ separations* within the Samaria corpus, and then provide a statistical estimate for the number of *authors* that could have created such an observation, taking into account possible detections and non-detections errors. Below, we provide a concise description of the two stages; for further details see the S1 File, Sections 1–2.

#### Stage I: Differentiating between authors

The algorithmic foundation of our approach was laid and verified on modern Hebrew handwriting [1]. Employing the algorithm on the ancient Arad ostraca [33] yielded a lower bound of four contemporaneous writers within a group of 18 inscriptions. The successful application of our approach on the noisy, deteriorated medium of the Arad ostraca indicated the potential for similar veins of research on other Hebrew Iron Age corpora.

**Fig 3:**
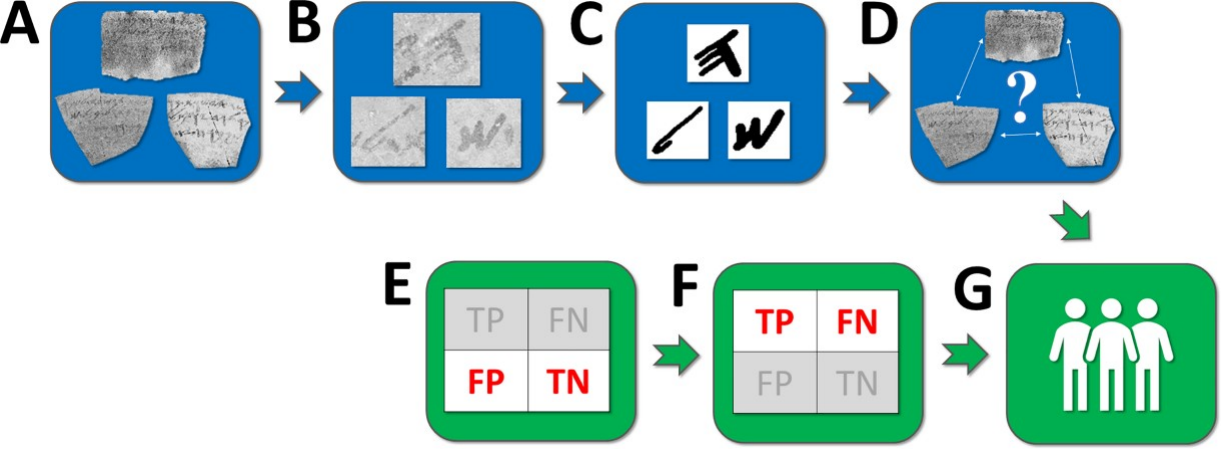
**Basic algorithmic flow**: **Stage I**: (**A**) scanned negatives of Samaria ostraca; (**B**) segmenting their characters; (**C**) restoring the characters and extracting features; (**D**) performing handwriting comparison. **Stage II**: (**E**) estimating False Positive and True Negative rates of the algorithm; (**F**) estimating True Positive and False Negative rates of the algorithm; (**G**) estimating the most likely number of scribes.

The algorithm comprises a sequence of three consecutive sub-steps (Fig 3A–3D), assuming digital images of the ostraca as its input (see S1 File, Section 1 for details, including certain enhancements with respect to [1], and up-to-date results on both modern handwriting and the Arad corpus; see also Tables B-C in S1 File), and operates on a character level. Throughout the article, by *character* we denote a particular instance of a given *letter* (e.g., there may be many characters, which are all occurrences of the letter *alep*).

- **Restoring characters** (based on [34]; cf. [35,36]). The image is segmented into (often noisy) characters that are restored via a semi-automatic reconstruction procedure. The purpose of the character restoration is to imitate a reed-pen’s movement using several manually sampled key-points on the grayscale input images. An optimization of the pen’s trajectory is performed for all intermediate sampled points. The restoration is conducted computationally via the minimization of an image energy functional, which considers the adherence to the original image, the smoothness of the stroke, as well as certain properties of the reed radius.
- **Extraction of characters’ features**. We adapted several well-known features, which describe aspects such as the character’s overall shape, the angles between strokes, the character’s center of gravity, as well as its horizontal and vertical projections (cf. [37]). The features, extracted from the reconstructed characters’ binary images, are: SIFT [38,39] (maximal curvature points of the strokes); Zernike [40,41] (representation of the overall shape related to the human visual system); DCT [42] (representation of the overall shape related to frequency analysis); Kd-tree [42,43] (mapping based on iterative “center of gravity” calculations); image projections [44] (horizontal and vertical histograms); and *L*_*1*_ and CMI [45,46,25] (both for character template matching). All these features were taken/adapted from the character recognition literature (however, not necessarily in historical documents setting), e.g., [44]. Subsequently, we exploit the pair-wise distances between all characters using all features, in order to define a vector representation of each character. As a result, the distance between two given characters is just a Euclidean distance between their vector representations.
- **Testing the null hypothesis *H***_***0***_ (for each pair of ostraca), that *two given inscriptions were written by the same author*. A corresponding p-value (*P*) is deduced, leveraging the data from the previous step. If *P*≤0.1, we reject *H*_*0*_ and accept the competing hypothesis of *two different authors*; otherwise we remain undecided. The following procedure was conducted: given two inscriptions and a particular letter-type (e.g., *bet*), cluster the corresponding instances from the two inscriptions, based on their vector representations. Then, quantify the adherence of the clusters to the original inscriptions, and calculate the probability of achieving such (or better) observation. This practice is repeated for each letter-type, with the *P*’s combined into a single value through Fisher's method [47].

The end product of Stage I is a table containing the *P* for all the pair-wise comparisons of ostraca. Naturally, although our algorithm declares *P*≤0.1 as a case of two distinct hands, it may also signify a false positive result. Empirically, this situation occurs with probability of *much less* than the expected 0.1 (i.e., our algorithm is “conservative”). Nevertheless, false separations do exist and might even dominate the results, e.g., if the number of writers is small. As will be seen in the Results section bellow, the Samaria corpus is characterized by a small number of separations (Table 1). Hence, an inevitable question arises: Is it plausible to assume that most of these separations are in fact false ones, with only a handful of writers present? Conversely, if some of the separations are true, *what is the most likely number of scribes in the Samaria corpus*?

**Table 1:** Comparison between different Samaria ostraca using Stage I of the algorithmic framework.

Text	2	5	6	7	8	12	14	16a	17a	18	19	20	21	22	24a	29	35	36	38	42	43	45	51	52	53	54	55	56	57	59	62
**2**			0.07	0.4		0.35	0.08	0.4	0.23	0.71	0.4		1		0.4		0.4						0.4		0.71	0.4	0.4	0.4			
**5**						0.6							0.4	0.16																	
**6**	0.07				0.4	0.61	0.09	0.07	0.12	0.23			0.2					1			1	0.95			0.19				1	1	0.6
**7**	0.4						0.03		0.6	0.27			0.16												0.27						
**8**			0.4			1	1						1									1			1						
**12**	0.35	0.6	0.61		1		0.9	0.62	0.7	0.94	0.45		0.48	0.41			0.4		1			0.91		0.6	0.35	0.39	0.91			0.6	
**14**	0.08		0.09	0.03	1	0.9		0.6	0.28	0.86	0.42		0.08	0.16	0.42		0.42				0.27	1	0.03		0.67	0.16	0.36	0.03		0.4	
**16a**	0.4		0.07			0.62	0.6		0.4	0.09			0.37	0.81								0.6			0.13		0.4				
**17a**	0.23		0.12	0.6		0.7	0.28	0.4		0.19	0.4		0.74	0.81	0.4		0.4					1	0.4		0.84	0.4	0.12	0.27			
**18**	0.71		0.23	0.27		0.94	0.86	0.09	0.19		0.58		0.25		1		1					0.6	0.6		0.15	0.91	0.61	0.6			
**19**	0.4					0.45	0.42		0.4	0.58			0.5	0.81											0.58						
**20**													0.91														0.4				
**21**	1	0.4	0.2	0.16	1	0.48	0.08	0.37	0.74	0.25	0.5	0.91		0.86	0.42	1	0.47		0.4	0.27		0.27	0.42		0.69	0.5	0.83	0.42			
**22**		0.16				0.41	0.16	0.81	0.81		0.81		0.86				0.42		0.16						0.81	0.81	0.81				
**24a**	0.4						0.42		0.4	1			0.42												1						
**29**													1														0.4				
**35**	0.4					0.4	0.42		0.4	1			0.47	0.42											0.4						
**36**			1																												
**38**						1							0.4	0.16																	
**42**													0.27														0.07				
**43**			1				0.27															0.27			0.6						
**45**			0.95		1	0.91	1	0.6	1	0.6			0.27								0.27				0.23					0.4	
**51**	0.4						0.03		0.4	0.6			0.42												0.6						
**52**						0.6																									
**53**	0.71		0.19	0.27	1	0.35	0.67	0.13	0.84	0.15	0.58		0.69	0.81	1		0.4				0.6	0.23	0.6			0.77	0.93	0.27		1	
**54**	0.4					0.39	0.16		0.4	0.91			0.5	0.81											0.77						
**55**	0.4					0.91	0.36	0.4	0.12	0.61		0.4	0.83	0.81		0.4				0.07					0.93						
**56**	0.4						0.03		0.27	0.6			0.42												0.27						
**57**			1																												
**59**			1			0.6	0.4															0.4			1						
**62**			0.6																			0.4			1						

A P≤0.1, highlighted in red, indicates writers’ separation established by our algorithm.

#### Stage II: Estimating the most likely number of authors

The estimation of the most likely number of scribes in the Samaria corpus necessitated the development of a new statistical framework. Its sub-steps are as follows (see Fig 3E–3G; for further details, see the S1 File, Section 2):

- **Estimating True Negative (TN) and False Positive (FP) rates** via “same writer” simulations.
- **Estimating True Positive (TP) and False Negative (FN) rates** via “different writer” simulations.
- **Estimating the most likely number of writers** within the corpus (via a Maximum Likelihood procedure).

Steps A and B assess the empirical probabilities for TP, TN, FP, and FN separations, i.e., the *confusion matrices*, in different configurations (see the S1 File, Section 2). Naturally, the simulations estimating the confusion matrices for a given corpus (in our case, Samaria) require an independent set of documents, preferably from approximately the same period, medium, language, and script. In order to be able to evaluate the percentage of false/true detections/misdetections of separate hands (i.e., TP, TN, FP, and FN) through Monte Carlo simulations, these inscriptions should be accompanied by pre-established separations between their authors. In this study, we consider the abovementioned Arad corpus [1,33], the richest among Hebrew Iron Age corpora, and the separations presented in the S1 File, Section 1, as the most suitable basis for our simulations. (Indeed, although at least a century and a half younger than the Samaria inscriptions, the Arad documents were written in the same script, using the same language, and utilizing the same medium–ink on clay; in fact, even the contexts of these corpora, mainly recording a supply of commodities, are rather similar). Thereafter, Step C estimates the empirical distributions of *separations*, considering scenarios of different *number of writers*. For instance, assuming that all the inscriptions were created by a single scribe, we conduct a Monte Carlo simulation in order to assess its corresponding conditional Probability Density Function (PDF), i.e., the probabilities of obtaining no separations for the whole corpus, a single separation, two separations, etc. Subsequently, assuming two writers in the corpus, we estimate another conditional PDF, and so on. In total, we estimate the conditional PDF's for scenarios ranging from a single writer to a number of writers equaling the number of inscriptions. A scenario maximizing the PDF value at the *observed number of separations*, provides us with the *MLE for the number of writers*. Moreover, under a confidence level of 1-α = 0.95, there may be other possible estimates for the number of writers.

## Results

Our algorithm was applied to 31 legible Samaria texts with sufficient textual information and a low curvature of text lines (namely, texts 2, 5, 6, 7, 8, 12, 14, 16a, 17a, 18, 19, 20, 21, 22, 24a, 29, 35, 36, 38, 42, 43, 45, 51, 52, 53, 54, 55, 56, 57, 59, and 62). In addition, eight texts were used for enriching the features’ statistics (11, 15, 17b, 33, 34, 40, 44, and 61; see the S1 File, Section 1). Note the double-sided ostraca 16, 17 and 24, with the *recto* denoted as “a,” and the *verso* denoted as “b.” All available letters with sufficient quantities were utilized: the Hebrew *bet*, *yod*, *lamed*, *mem*, *nun*, *resh*, *shin*, and *taw*. In total, 293 legible characters were restored, based upon computerized images of the inscriptions.

The complete results of Stage I of our framework, applied to the Samaria corpus, are summarized in Table 1. The ostraca numbers head the rows and columns of the table, and the intersection cells provide the comparisons’ p-values. The cells with *P*≤0.1 are marked in red, indicating that the two ostraca are considered to be written by different authors. We reiterate that when *P*>0.1, we cannot claim that they were written by a single author; in such a case our algorithm remains agnostic.

As seen in Table 1, most of the Samaria ostraca pairs could not have been compared (gray cells), due to insufficient letter statistics. This is caused by the brevity of the inscriptions, containing a very low number of legible restored characters for each text (9.5 characters on average). Nevertheless, 138 comparisons were performed, yielding **10 separations**.

**Fig 4:**
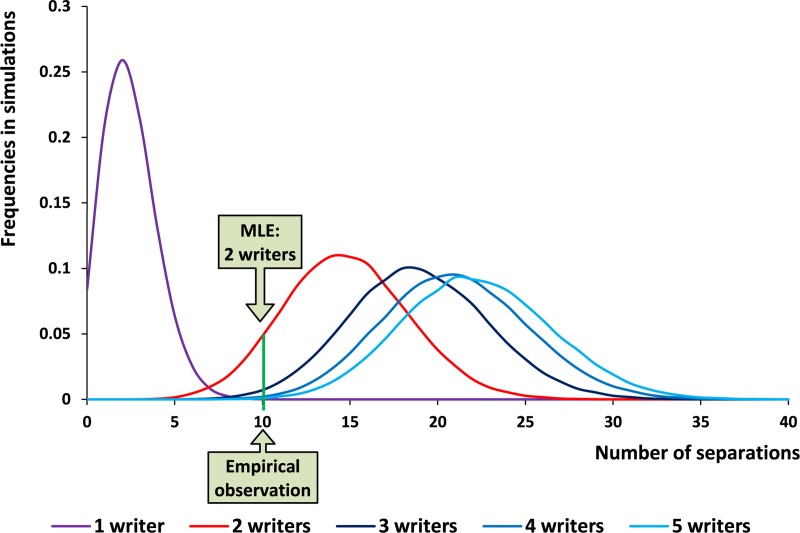
Conditional PDF for the number of separations in our ostraca sample, with different simulated number of writers. The MLE is two authors, based on 10 observed separations.

In order to assess the most likely number of writers, Stage II of the algorithm was applied. A summary of the results can be seen in Fig 4. It depicts the conditional PDFs for different number of writers' scenarios, ranging from a single to up to five scribes (our simulations included scenarios of 1–31 writers; only graphs for 1–5 writers are presented). It can be seen that provided the previously obtained 10 separations, **the most likely estimate for the number of writers in Samaria is two**. Furthermore, out of all tested scenarios, two is the only valid estimate for the number of writers under a confidence level of 1-α = 0.95.

The confusion matrices of Stage II, Step B were based on the separation statistics, stemming from the application of Stage I on the Arad corpus. This application (utilizing a threshold of *P* = 0.1) may have theoretically focused on data producing “excessively” significant results, while restricting the number of potentially false outcomes. In order to rule out this possibility, another simulation, in a less restrictive mode (employing a threshold of *P* = 0.2 for Arad only), was conducted. Our main results, including the MLE, were upheld even in this setting (see the S1 File, Section 2, as well as Fig A in S1 File and Table G in S1 File, for additional details).

## Discussion

The maximum likelihood estimate of two writers, obtained in this research, seems to shed light on the administrative apparatus in the kingdom of Israel. As mentioned above, the 31 tested Samaria ostraca, spanning a maximum of seven years (assuming a single monarch; cf. *8* which calculates one year, more below), contain various documentation characteristics (i.e., year, commodity, name of person, clan and toponym). We tried to find an interrelation between the two writers and these characteristics. Various clustering algorithms (e.g., SVM-clustering, max-cut, k-means, PCA-based methods) were applied on both the feature vector representations and final p-value tables. However, the clustering results were inconsistent and did not create even an approximate division according to any particular characteristic (e.g., year, type of commodity, etc.). Moreover, a close examination of the characteristics detailed in Table D in S1 File vs. the separations obtained in Table 1, reveals that a clear-cut division of the ostraca is impossible for any characteristic (e.g., Ostraca 6 and 14 are separated by the algorithm despite mentioning the same commodity, wine; Ostraca 16 and 18 are separated although both mention oil). In other words, a hypothetical split of the inscription into two groups according to any particular characteristic cannot be achieved. As a result, and in light of the short span of regnal years mentioned (9–15, or a single year if one follows [8]), we suggest that the **two writers were contemporaneous, and performed similar duties**.

Furthermore, although the ostraca originated from various locations in the highlands around the capital and mention different clans in the region (see Fig 2), the fact that they were written by only two individuals seems to indicate that **the scribes were located in Samaria** rather than in the countryside. While we cannot rule out the possibility that the two scribes were traversing the countryside, documenting shipments on demand (cf. the “wandering scribes” of the Amarna tablets, offering their services to rulers of city-states of Late Bronze Canaan; see [48]) we see such a possibility as less plausible in the case of mundane activities (shipment of agricultural goods to differ from diplomatic correspondence) and an organized and well-governed kingdom. Note that the only contemporaneous corpus–that of Kuntillet Ajrud in the remote northern Sinai Desert [49]–is also related to the royal administration of the kingdom of Israel. This seems to attest to the existence of a centralized bureaucratic apparatus in Samaria in particular and in the Northern Kingdom in general.

One may ask why the Samaria ostraca do not refer to earlier or later years of Israelite monarchs. To answer, we first note that the ostraca were found out of stratigraphic context, in a fill prepared for the construction of the "Ostraca House" [8] and possibly even beyond [9], meaning that there is no way to trace their original provenance. Theoretically, they may represent one collection of records, while other collections may have been disposed of in other places. In any event, the dating of the Samaria ostraca can be narrowed down by taking the year 15 mentioned in some of them as being attributed to the rule of a specific king [6]. According to the biblical account, correlated with Assyrian records, only five kings ruled in Samaria for periods of 15 years or longer (Ahab 871/873-852 BCE; Jehu 842–814 BCE; Jehoahaz 817–800 BCE; Joash 800–784 BCE; and Jeroboam II 788–747 BCE (dates of Jehoahaz and Jeroboam II include coregencies). Since proto-Canaanite script (which antedates the Hebrew script) still appears in the 9^th^ century [50,51], the days of Joash and Jeroboam II are the most likely. Judging from the prosperity of the kingdom, the latter option is the most plausible, though the fact that the latest year referred to is 15 may point to the former (Jeroboam II ruled for over 40 years, while his father Joash reigned for 16 years). One may hypothesize that the Samaria ostraca represent an important phase in the bureaucracy of the kingdom. During the days of Joash or the first years of Jeroboam II, Hebrew writing had already been sufficiently developed to enable recording on ostraca; a while later, during the peak prosperity of the kingdom, the system could have changed to a more efficient recording system, perhaps using papyri.

As mentioned above (see also [52]), there is a high level of standardization in the format of the Samaria texts, which may support the proposed bureaucratic apparatus, perhaps even administrative centralization. Nevertheless, at some point between the years 10 and 15 a change in the documentation formula occurred: ostraca belonging to year 9 or 10 contain commodity type and neglect the clan feature, whereas ostraca belonging to year 15 neglect the commodity type and contain the clan name. As already noted, we could not associate years 9–10 with one scribe and 15 with the other. Therefore, since there are two contemporaneous scribes, this change may be attributed to a new/different administration directive, rather than to the scribes’ preferences. In addition, there is a noticeable increase in the number of inscriptions pertaining to year 15. Explicitly, there are 9 inscriptions bearing the year 9; 14 inscriptions bearing the year 10; and 29 inscriptions bearing the year 15. If the sample we have is representative, this may indicate increased activity during the later years of the given monarch/s.

Another notable consequence of the current research pertains to the field of paleography. Apart from the two Samaria scribes, the only significant pieces of evidence for writing with ink on clay-sherds in the Northern Kingdom are two inscriptions from Beit-Shean (20 km from Samaria; see [53]) and the inscriptions from Kuntillet Ajrud in the Sinai Desert (~250 km from Samaria; see [49]). The latter site is short-lived and small (a single building), therefore, the number of scribes there must have been restricted. Archaeologically (ceramic evidence for Beth-Shean and Kuntillet Ajrud) and paleographically (Samaria), these inscriptions all date to the same period. They represent a handful of scribes and hence–contra to conventional wisdom–it is doubtful if one can construct a reliable paleographic system based on these finds.

Contrasting the epigraphic evidence presented above, covering the territory of the kingdom of Israel in the early 8^th^ century BCE, with the level of literacy in the kingdom of Judah ca. 600 BCE based on our study of the Arad ostraca ([1], acknowledging the different natures of the two corpora–military correspondence versus receipts of shipments of agricultural commodities), an interesting tendency appears. On the one hand, we observe just two scribes within the large Samaria ink ostraca corpus of the flourishing Northern Kingdom’s capital, with very little supporting evidence of writing skills from other sites in the realm. This may hint that during this period literacy was, to some extent, restricted to the royal court (note that Kuntillet Ajrud is apparently also related to the kingdom's administration). On the other hand, in a small desert outpost far from the center of Judah of the late 7^th^ century, at least six contemporaneous writers are attested [1]. Indeed, thriving scribal activity in Judah is demonstrated by other, contemporaneous corpora, such as those from Lachish [54], Horvat ‘Uza, Horvat Radum [55] and Tel Malhata [56], as well as individual ostraca scattered far and wide across the kingdom, e.g., the Ophel in Jerusalem [57], Mezad Hashavyahu on the coast [58], and Nahal Yarmut in the Shephelah [59]. In other words, over the course of the century and a half or two centuries that separate the two corpora, we observe development from a writing milieu centered mainly around the royal court to a broad proliferation of literacy.

## Supporting information

S1 File Supplementary material. (PDF)
