# Supplementary material for: Algorithmic handwriting analysis of the Samaria inscriptions illuminates bureaucratic apparatus in biblical Israel
Source: PLoS One. 2020 Jan 22;15(1):e0227452. doi: 10.1371/journal.pone.0227452 (PMC6975535; doi:10.1371/journal.pone.0227452)
Supplement: S1 File — (PDF) [file pone.0227452.s001.pdf]

Supplementary Material

Section 1: Differentiating Between Authors

Introduction

The goal of this algorithm is to distinguish between authors of different inscriptions. The previous version of this method was presented in [1]. The algorithm takes into account the poor state of preservation of Iron Age ostraca, and the high variance of their cursive texts, in order to estimate the probability that two given inscriptions were written by the same author. If the probability is lower than a pre-selected threshold, we consider the inscription to be written by two different authors.

The input for our system is the digital images of the inscriptions, obtained by scanning the negatives from the original excavation, kept at the Harvard Semitic Museum (ostraca tend to fade upon unearthing, and even modern image acquisition techniques such as described in [18–24], would doubtfully be of use a century after the excavations); see examples on Fig 1. The algorithm involves two preparatory stages, leading to a third step that estimates the probability that two given inscriptions were written by the same author. All the stages are fully automatic, with the exception of the first, semi-automatic, preparatory step. The basic steps of the algorithm are:

A. **Restoring characters** via approximation of their composing strokes, represented as a spline-based structure, and estimated by an optimization procedure (for further details see sub-section 1A).

B. **Feature Extraction and Distance Calculation**: creation of feature vectors describing various aspects of the characters (e.g., angles between strokes and character profiles) and calculating the distance (similarity) between characters (see sub-section 1B).

C. **Testing the hypothesis that *two given inscriptions were written by the same author***. Upon obtaining a suitable p-value (the significance level of the test, denoted as *P*), we reject the hypothesis of a single author and accept the competing proposition of two different authors; otherwise we remain undecided (see sub-section 1C).

In the current paper, several changes were carried out with respect to the original algorithm reported in [1]. The main alterations (discussed in more details in sub-section 1C) are: lowering the *P* threshold to 0.l; in sub-step 2, k-medoid replacing the k-mean clustering algorithm; the formula in sub-step 3 was changed in order to better represent the non-homogeneity of both of the classes/inscriptions; the calculations in sub-step 4 was altered with the parameter NC now taking into account all the potential clustering outcomes, resulting in more accurate *P* calculations. Accordingly, the "Experiment details and results" sub-section includes updated outcomes for modern documents and the ancient Arad corpus; as well as entirely new experimental results for the ancient Samaria corpus (see further details below).

Description of the Algorithm

A. Character Restoration

The state of preservation of most ostraca is poor at best. After more than two and a half millennia buried in the ground, the inscriptions are often blurry, partially erased, cracked, and stained. Yet, in order to analyze the script, clear black and white ("binary") images are required. Theoretically, such depictions of the inscriptions do exist, in the form of manually created facsimiles (drawings of the ostraca), created by epigraphic experts. However, these have been shown to be influenced by the prior knowledge and assumptions of the epigrapher [45]. A potential solution for this problem could have been provided by automatic binarization procedures from the domain of image

processing. Unfortunately, in our experimentations, various binarization methods produced unsatisfactory results [25].

Therefore, we chose a semi-automatic approach for character restoration. Restoring a character is equivalent to reconstructing its strokes, which are the character's building blocks, and then combining them. Accordingly, henceforth we will discuss the problem of stroke restoration rather than complete character reconstruction. Stroke restoration aims at imitating the reed-pen's movement using several manually sampled key-points. An optimization of the pen's trajectory is performed for all intermediate sampled points, taking into account information from the noisy character image. A short mathematical description of the procedure follows. For more details and analysis see [34].

A stroke could be referred to as a two-dimensional piecewise smooth curve $\left(x(t), y(t)\right)$, depending on the parameter $t \in [a,b]$. However, such a representation ignores the stroke's thickness, which is related to the stance of the writing pen towards the document (in our case – potsherd) and to the characteristics of the pen itself. In the case of Iron Age Hebrew, it is well accepted that the scribes used reed pens, which have a flat, rather than pointed nib. This fact makes the writing thickness even more essential to the stroke restoration process. Therefore, we define the *stroke* as a set-valued function:

$$S(t) = \left\{ (p,q) \mid \left(p - x(t)\right)^2 + \left(q - y(t)\right)^2 \le r(t)^2 \right\} \quad t \in [a,b],$$

where $x(t)$ and $y(t)$ represent the coordinates of the center of the pen at t, and $r(t)$ stands for the radius of the pen at t (for additional details, see [34]). The corresponding *stroke-curve* is thus:

$$\gamma(t) = \left(x(t), y(t), r(t)\right) \quad t \in [a,b],$$

while the *skeleton of the stroke* will accordingly be the curve:

$$\beta(t) = \left(x(t), y(t)\right) \quad t \in [a,b].$$

We note that our model of a written stroke is an approximation, since in reality the top of the reed pen was not necessarily a perfect circle. Furthermore, the result may even have had different "local" radii, depending on the tilt of the reed along the stroke.

Following the idea of minimizing an energy functional [35,36], we produce a spline-based reconstruction of a stroke with respect to a given image $I(p,q)$ ($(p,q) \in [1,N] \times [1,M]$). This reconstructed stroke $S^*(t)$ is defined as corresponding to the stroke-curve $\gamma^*(t)$, minimizing the following functional:

$$F\left[\gamma(t)\right] = c_1 \int_a^b \frac{G_I(t)}{r_{(t)}{}^2} dt + c_2 \int_a^b \frac{1}{\sqrt{r_{(t)}}} dt + c_3 \sum_{j=0}^{J-1} \int_{t_j+\varepsilon}^{t_{j+1}-\varepsilon} \left|K\left(\dot{x}, \dot{y}, \ddot{x}, \ddot{y}\right)\right| dt$$

$$\gamma^*(t) = \arg\min_{\gamma(t)} F\left[\gamma(t)\right],$$

where $G_I(t) = \sum_{(p,q) \in S(t)} I(p,q)$ is the sum of the gray level values of the image I inside the disc $S(t)$; $\gamma(t_j) = \left(x(t_j), y(t_j), r(t_j)\right) \quad j = 0,...,J$ are manually sampled points on the stroke-curve $\gamma(t)$, with respect to the natural parameter t; $\dot{x}$, $\ddot{x}$ and $\dot{y}$, $\ddot{y}$ denote the first and second derivatives of *x* and *y*; $K\left(\dot{x}, \dot{y}, \ddot{x}, \ddot{y}\right) = \left(\dot{x}\ddot{y} - \dot{y}\ddot{x}\right) / \left(\dot{x}^2 + \dot{y}^2\right)^{3/2}$ stands for the curvature of the skeleton of the stroke $\beta(t)$; $0 < c_1, c_2, c_3, \varepsilon \in \mathbb{R}$ are parameters, set to $c_1 = 2, c_2 = 2000, c_3 = 50, \varepsilon = 0.01$ in our experiments.

The reconstruction is subject to boundary conditions at: a) the beginning and end of strokes; b) intersections of strokes; c) significant extremal points of the curvature; d) points with no traces of ink. These conditions ought to be supplied by manual sampling.

The energy minimization problem described above is solved by performing Gradient Descent iterations on a cubic spline representation of the stroke (for more details see [34]; also see examples

in Fig 3). The end product of the reconstruction is a binary image of the character, incorporating all its strokes.

B. Feature Extraction and Distance Calculation

Commonly, automatic comparison of characters relies upon features extracted from the characters' binary images. In this study, we adapted several well-established features from the domains of Computer Vision and Document Analysis. These features refer to aspects such as the character's overall shape, the angles between strokes, the character's center of gravity, as well as its horizontal and vertical projections. Some of these features correspond to characteristics commonly employed in traditional paleography [37].

The feature extraction process includes a preliminary step of the characters' standardization. The steps involve rotating the characters according to their line inclination, resizing them according to a pre-defined scale, and fitting the results into a padded (at least 10% on each side) square of size $a_L \times a_L$ (with $L = 1,...,22$ the index of the alphabet letter under consideration). On average, the resized characters were 300 by 300 pixels.

Subsequently, the proximity of two characters can be measured using each of the extracted features, representing various aspects of the characters. For each such feature, a *different* distance function is defined (later these distances are combined to create a vector representation of each character; see discussion below).

Table A provides a list of the features and distances we employ, along with a description of their implementation details. Some of the adjustments (e.g., replacement of the L_2 norm with the L_1 norm) were required due to the large amount of noise present in our medium.

Table A. Features and distances utilized by the writers' separation algorithm.

**Feature** [reference]	**Feature implementation details**	**Distance implementation details**
**SIFT** [38]	For each character j, we use the normalized SIFT descriptors $\vec{d}_i \in \mathbb{R}^{128}$ (with $\left\|\vec{d}_i\right\|_2 = 1$) and the spatial locators $\vec{l}_i \in [1, a_L]^2$ for at most 40 significant key points $k_i = \left(\vec{d}_i, \vec{l}_i\right)$, according to the original SIFT implementation. The resulting feature is a set $f_j^{SIFT} = \left\{k_i\right\}_{i=1}^{40}$.	The distance between f_1^{SIFT} and f_2^{SIFT} is determined as follows:<br>1. For each key point $k_i^1 \in f_1^{SIFT}$, find a matching key point $m_i^2 \in f_2^{SIFT}$ s. t. $m_i^2 = \underset{\left(d_j^2, l_j^2\right) \in f_2^{SIFT}}{\arg\min}\ dist(k_i^1, k_j^2)$; where $dist(k_i^1, k_j^2) = \arccos\left(\left\langle d_i^1, d_j^2 \right\rangle\right) \cdot \left\| l_i^1 - l_j^2 \right\|_2^2$. Thus, our definition enhances the original SIFT distance by adding spatial information.<br>2. The one-sided distance is $D_{SIFT}^{1,2} = \underset{i}{median}\left\{dist(k_i^1, m_i^2)\right\}$.<br>3. The final distance is $D_{SIFT}(1,2) = \frac{D_{SIFT}^{1,2} + D_{SIFT}^{2,1}}{2}$.
**Zernike** [40]	An off-the-shelf [41] implementation was used. Zernike moments up to the 5th order were calculated.	$D_{Zernike}$ is the L_1 distance between the Zernike feature vectors.
**DCT**	Standard MATLAB implementation was used.	D_{DCT} is the L_1 distance between the DCT feature vectors.
Kd-tree [42]	An off-the-shelf [43] implementation was used. Both orders of partitioning are employed (first height, then width and vice-versa)	$D_{Kd-tree}$ is the L_1 distance between the Kd-tree feature vectors.
**Image projections** [44]	The implementation results in cumulative distribution functions of the histogram on both axes.	D_{Proj} is the L_1 distance between the projections' feature vectors; this is similar to the Cramér–von Mises criterion (which uses L_2 distance).
**L1**	Existing character binarizations.	D_{L1} is the L_1 distance between the character images.
**CMI** [45,46,25]	Existing character binarizations, with values in $\{0,1\}$.	The CMI computes a difference between the averages of the foreground and the background pixels of $\mathcal{I}$, marked by a binary mask M, $CMI(M, \mathcal{I}) = \mu_1 - \mu_0$, where:<br>$\mu_k = mean\{\mathcal{I}(p,q) \mid M(p,q) = k\} \quad k = 0,1$<br>In our case, given character-binarizations B_1, B_2, the one-sided distance is $D_{CMI}^{1,2} = 1 - CMI(B_1, B_2)$.<br>The final distance is $D_{CMI}(1,2) = \frac{D_{CMI}^{1,2} + D_{CMI}^{2,1}}{2}$.

After the features are extracted, and the distances between the features are measured, a combination of the various distances is required. In [1], a new combination technique was proposed. The main idea was to consider the distances of a given character from *all the other characters*, with respect to *all of the features* under consideration. I.e., two characters closely resembling each other ought

to have similar distances when compared to all other characters. Namely, they will both be at small distances from similar characters, and large distances from dissimilar characters. This observation leads to a notion of a *generalized feature vector*.

The generalized feature vector is defined by the following procedure (for each letter $L = 1, ..., 22$ in the alphabet). First, we define a *distance matrix* for each feature. For example, the SIFT distance matrix is:

$$U_{SIFT} = \begin{pmatrix} D_{SIFT}(1,1) & \cdots & D_{SIFT}(1, J_L) \\ \vdots & \ddots & \vdots \\ D_{SIFT}(J_L, 1) & \cdots & D_{SIFT}(J_L, J_L) \end{pmatrix} = \begin{pmatrix} - & \vec{u}_{SIFT}^{1} & - \\ & \vdots & \\ - & \vec{u}_{SIFT}^{J_L} & - \end{pmatrix},$$

where J_L represents the total number of characters; $D_{SIFT}(i, j)$ is the SIFT distance between characters *i* and *j*; while $\vec{u}_{SIFT}^{i} = (D_{SIFT}(i,1) \cdots D_{SIFT}(i, J_L))$ is the vector of SIFT distances between the character *i* and all the others.

In addition, we denote the standard deviation of the elements of the matrix U_{SIFT} by $\sigma_{SIFT} = std\left\{D_{SIFT}(i, j) \,|\, (i, j) \in \{1,..., J_L\} \times \{1,..., J_L\}\right\}$. Matrices of all the other features ($U_{Zernike}$, U_{DCT}, and so forth) and their respective standard deviations ($\sigma_{Zernike}$, σ_{DCT}, etc.) are calculated in a similar fashion.

Eventually, each character k is represented by the following vector (of size $7 \cdot J_L$), concatenating the respective normalized row vectors of the distance matrices:

$$\vec{u}_k = \left(\frac{\vec{u}_{SIFT}^{k}}{\sigma_{SIFT}} \,||\, \frac{\vec{u}_{Zernike}^{k}}{\sigma_{Zernike}} \,||\, \frac{\vec{u}_{DCT}^{k}}{\sigma_{DCT}} \,||\, \frac{\vec{u}_{Kd-tree}^{k}}{\sigma_{Kd-tree}} \,||\, \frac{\vec{u}_{Proj}^{k}}{\sigma_{Proj}} \,||\, \frac{\vec{u}_{L1}^{k}}{\sigma_{L1}} \,||\, \frac{\vec{u}_{CMI}^{k}}{\sigma_{CMI}} \right) \in \mathbb{R}^{7 \cdot J_L}$$

In this fashion, each character is described by the degree of its kinship to all of the characters, using all the various features.

Finally, the distance between characters i and j is calculated according to the Euclidean distance between their generalized feature vectors:

$$chardist(i, j) = \left\| \vec{u}_i - \vec{u}_j \right\|_2 .$$

The main purpose of this distance is to serve as a basis for clustering at the next stage of the analysis.

C. Hypothesis Testing

At this stage we address the main question raised above: "*What is the probability that two given texts were written by the same author*?" Commonly, similar questions are addressed by posing an *alternative* null hypothesis H_0 and attempting to reject it. In our case, for each pair of ostraca, the H_0 is: *both texts were written by the same author*. This is performed by conducting an experiment (detailed below) and calculating the probability ($P \in [0,1]$) of affirmative answer to H_0. If this event is unlikely ($P \leq 0.1$; note this is a change of threshold with respect to [1]), we conclude that the documents were written by two different individuals (i.e., reject H_0). On the other hand, if the occurrence of H_0 is probable ($P > 0.1$), we remain agnostic. We reiterate that in the latter case we cannot conclude that the two texts were in fact written by a single author.

The experiment, which is designed to test H_0, is comprised of several sub-steps (for additional details see [1]):

1. **Initialization**: We begin with two sets of characters of the same letter type (e.g., *alep*), denoted *A* and *B*, originating from two different texts.
2. **Character clustering**: The union $A \cup B$ is a new, unlabeled set. This set is clustered into two classes, labeled I and II, using a brute-force (and not heuristic) implementation of k-medoids

(k=2; note the clustering is not k-means as stated in [1]). The clustering utilizes the generalized feature vectors of the characters, and the distance *chardist*, defined above.

3. **Non-homogeneity (NH) of the clustering**: The observed difference between the uniformity of the clustering results to the two original sets, *A* and *B*, is calculated as follows:

$$NH = NH_I = \left| \frac{\#(A \cap I)}{\# A} - \frac{\#(B \cap I)}{\# B} \right|,$$

with # denoting a cardinality of a given set. It is easy to verify that the non-homogeneity score is well-defined, i.e., it is invariant to swap between I and II, $NH = NH_I = NH_{II}$. Note that this symmetry was enabled by a definition change with respect to [1].

4. **Counting valid combinations**: We consider all the possible divisions of $A \cup B$ into two classes i and ii. The number of such valid combinations is denoted by NC. In fact, $NC = 2^{\#(A \cup B)} - 2$, since all the assignments of the characters to classes i and ii are considered, except for labeling all the characters as a single class. Note that this valid combinations' calculation is more inclusive than in [1].

5. **Significance level calculation**: The p-value is calculated as:

$$P = \frac{\#\{i \mid NH_i \geq NH\}}{NC} \quad .$$

I.e., P is the proportion of valid combinations with at least the same observed non-homogeneity. This is analogous to integrating over a tail of a probability density function.

The rationale behind this calculation is based on the scenario of *two authors* (negation of H_0). In such a case, we expect the k-medoids clustering to provide a sound separation of their characters, i.e., I and II would closely resemble A and B (or B and A). This would result in NH being

close to 1. Furthermore, the proportion of valid combinations with $NH_i \geq NH$ will be meager, resulting in a low P. Therefore, the H_0 hypothesis would be justifiably rejected.

In the opposite scenario of a *single author*:

- If a sufficient number of characters is present, there is an arbitrary low probability of receiving clustering results resembling A and B. In a common case, the NH will be low, which will result in high P.
- Alternatively, if the number of characters is low, the clustering may result in a high NH by chance. However, in this case NC would be low, and the P would remain high.

Either way, typically in this scenario we will not be able to reject the H_0 hypothesis.

Notes:

- We assume that each given text was written by a single author. If multiple authors wrote the text, both H_0 and its negation should be altered. We do not cover such a case.
- The definition of P in sub-step 5 results in $P > 0$.
- Not every text provides a sufficient amount of characters for every type of letter in the alphabet. In our case, we do not perform comparisons for sets A and B such that: $(\#A = 1) \& (\#B \leq 6)$ or $(\#B = 1) \& (\#A \leq 6)$ or $(\#A = 2) \& (\#B = 2)$.

As specified, sub-steps 1-5 are applied to one specific letter of the alphabet (e.g., *alep*), present (in sufficient quantities) in the pair of texts under comparison. However, we can often gain additional statistical significance if several different letters (e.g., *alep*, *he*, *waw*, etc.) are represented in the compared documents. In such circumstances, several independent experiments are conducted (one for each letter), resulting in corresponding *P*'s. We combine the different values into a single P

using the well-established Fisher method [47]. This end product represents the probability that H_0 is true based on all the evidence at our disposal.

Experimental details and results

Our experiments were conducted on three datasets. The first is a set of samples collected from contemporary writers of Modern Hebrew, described and provided in [1]. This dataset allowed us to test the soundness of our algorithm. It was not used for parameter-tuning purposes, however, as the algorithm was kept as parameter-free as possible. The second dataset contained information from various Arad Ancient Hebrew ostraca, dated to ca. 600 BCE, described in detail and provided in [1]. The third dataset includes data from the corpus of Samaria, the capital of the Israelite kingdom. These ostraca were probably produced in the first half of the 8th century BCE. This dataset is provided in [17].

<u>Modern Hebrew script experiment</u>

The handwriting of 18 individuals $i = 1,...,18$ was sampled. Each individual filled in a modern Hebrew alphabet table consisting of ten occurrences of each of the 22 letters in the alphabet (the number of letters in the alphabet are the same in both ancient and modern Hebrew). These tables were scanned, and their characters were segmented; see [1] for details and the dataset.

From this raw data, a series of “simulated” inscriptions were created. Due to the need to test both same-writer and different-writer scenarios, the data for each writer was split. Furthermore, in order to imitate a common situation in the ancient corpora, where the scarcity of data is prevalent, each simulated inscription used only 3 letters (i.e., 15 characters; 5 characters for each letter). In total, 252 inscriptions were “simulated” in the following manner:

- All the letters of the alphabet except for *yod* (as it is too small to be considered by some of the features), were split randomly into 7 groups (3 letters in each group) $g = 1, \ldots, 7$: *gimel, het, resh*; *bet, samek, shin*; *dalet, zayin, ayin*; *tet, lamed, mem*; *nun, sade, taw*; *he, pe, qop*; *alep, waw, kap*.
- For each writer i, and each letter belonging to group g, 5 characters were assigned into simulated inscription $S_{i,g,1}$, with the rest assigned to $S_{i,g,2}$.

In this fashion, for constant *i* and *g*, we can test if our algorithm arrives at wrong rejection of H_0 for $S_{i,g,1}$ and $S_{i,g,2}$ (FP = "False Positive" error; 18 writers and 7 groups producing 126 tests in total). Additionally, for constant *g*, $1 \le i \ne j \le 18$, and $b, c \in \{1, 2\}$, we can test if our algorithm fails to correctly reject H_0 for $S_{i,g,b}$ and $S_{j,g,c}$ (FN = "False Negative" error; $\frac{18\text{x}17}{2}\text{x}7\text{x}2\text{x}2 = 4284$ tests in total).

The results of the modern Hebrew script experiment are summarized in Table B. It can be seen that in modern context, the algorithm yields reliable results in more than 95% of the cases (with 4.76% of FP and 2.66% FN error rates). These results demonstrate the soundness of our algorithmic sequence. In fact, taking into account the 0.1 threshold, the empirical error rates may indicate a "conservative" *P* estimation.

Table B. Results of the Modern Hebrew experiment

**Group of letters** (corresponding to *g*-index of simulated inscriptions)	**False Positive** (FP out of all same-writer comparisons)	**False Positive %** (FP out of all same-writer comparisons)	**False Negative** (FN out of all different-writer comparisons)	**False Negative %** (FN out of all different-writer comparisons)
gimel, het, resh	0 / 18	0	7 / 612	1.14
bet, samek, shin	1 / 18	5.56	5 / 612	0.82
dalet, zayin, ayin	2 / 18	11.11	30 / 612	4.9
tet, lamed, mem	0 / 18	0	17 / 612	2.78
nun, sade, taw	1 / 18	5.56	3 / 612	0.49
he, pe, qop	0 / 18	0	17 / 612	2.78
alep, waw, kap	2 / 18	5.56	35 / 612	5.72
**Total**	**6 / 126**	**4.76**	**114 / 4284**	**2.66**

Arad ancient Hebrew script experiment

This experiment addressed ostraca from the Arad fortress, located on the southern frontier of the kingdom of Judah. The inscriptions were composed during the span of a few years, ca. 600 BCE, and consist of army correspondence and documentation [33].

The texts under examination are 16 Arad Ostraca, Nos. 1, 2, 3, 5, 7, 8, 16, 17, 18, 21, 24, 31, 38, 39, 40 and 111. Ostraca 17 and 39 contain writing on both sides of the potsherd and were treated as separate texts (17a and 17b; 39a and 39b), resulting in 18 texts under examination. As we go to press, we have not yet obtained data from the newly-discovered *verso* side of Arad Ostracon 16 (see [20,21]).

The seven letters we utilized were: *alep, he, waw, yod, lamed, shin* and *taw*, as they were the most prominent and simple to restore. In total, 427 characters were restored. For additional details and a complete dataset of the characters, see [1]. The results obtained by comparing the Arad ostraca are summarized in Table C.

As can be seen from the table, 44 separations out of 149 comparisons were achieved, with the p-values as low as ~$2x10^{-6}$. Additionally, we can observe two pair-wise distinct "quintuplets" of texts: I) **7, 18, 24, 31** and **38**; II) **16, 18, 24, 31** and **38**. In other words, if the five pair-wise distinct authorships are indeed true, then at least five different hands produced the corpus of Arad inscriptions. The existence of two such combinations indicates the high probability that the corpus indeed contains at least five different authors (the probability of obtaining at least one pair-wise distinct "quintuplet" of texts on a random graph with a configuration similar to Arad, with edge probability of 0.1, is 1×10^{-7}). It will be stressed that the separated inscriptions 31 and 38 contain lists of names, and their authors were most likely located at the tiny fort of Arad itself. This implies the composition by writers who were not professional scribes; for additional discussion see [1].

Table C. Comparison between different Arad texts. A P≤0.1 highlighted in red, indicates rejection of "single writer" hypothesis, hence accepting a "two different authors" alternative. Note that Ostraca 17 and 39 contain writing on both sides of the sherd (marked as "a" and "b").

Text	1	2	3	5	7	8	16	17a	17b	18	21	24	31	38	39a	39b	40	111
1		0.23	0.75	0.86	0.29	0.25	0.45	0.29	0.64	0.01	0.71	0.01	0.03	0.60	0.41	0.21	0.02	0.45
2	0.23		0.28	0.09	0.11	0.85	0.27	0.14	0.90	0.09	0.02	0.12	0.01	0.38	0.70	0.01	0.06	0.86
3	0.75	0.28		0.85	0.11	0.68	0.81	0.47	0.99	0.03	0.55	0.80	0.51	0.12	0.99	0.91	0.07	0.69
5	0.86	0.09	0.85		0.87	0.31	0.46	0.47	0.21	0.06	0.63	0.14	0.03	0.13	0.17	0.42	0.27	0.46
7	0.29	0.11	0.11	0.87		0.22	0.81	0.16	3e-3	3e-3	0.36	4e-3	1e-3	0.01	0.13	0.73	0.03	0.01
8	0.25	0.85	0.68	0.31	0.22		0.65		0.03	0.06	0.41	2e-4	0.63	1.00	0.84	0.40	0.90	0.93
16	0.45	0.27	0.81	0.46	0.81	0.65		0.60	0.77	0.01	0.49	6e-4	1e-3	0.04	0.26	0.01	0.01	0.55
17a	0.29	0.14	0.47	0.47	0.16		0.60			0.36	0.98	0.23	0.54		1.00	0.81	0.42	1.00
17b	0.64	0.90	0.99	0.21	3e-3	0.03	0.77			0.15	0.12	0.18	0.24		0.85	0.69	0.15	0.92
18	0.01	0.09	0.03	0.06	3e-3	0.06	0.01	0.36	0.15		5e-5	7e-4	0.02	0.03	0.60	0.85	0.21	0.57
21	0.71	0.02	0.55	0.63	0.36	0.41	0.49	0.98	0.12	5e-5		0.04	5e-4	0.77	0.10	2e-4	0.02	0.12
24	0.01	0.12	0.80	0.14	4e-3	2e-4	6e-4	0.23	0.18	7e-4	0.04		2e-6	2e-3	0.53	0.25	7e-6	0.43
31	0.03	0.01	0.51	0.03	1e-3	0.63	1e-3	0.54	0.24	0.02	2e-3	2e-6		0.07	0.32	0.94	0.39	0.68
38	0.60	0.38	0.12	0.13	0.01	1.00	0.04			0.03	0.77	5e-4	0.07		0.37	0.81	0.46	0.58
39a	0.41	0.70	0.99	0.17	0.13	0.84	0.26	1.00	0.85	0.60	0.10	0.53	0.32	0.37		0.93	0.01	0.73
39b	0.21	0.01	0.91	0.42	0.73	0.40	0.01	0.81	0.69	0.85	2e-4	0.25	0.94	0.81	0.93		0.58	0.28
40	0.02	0.06	0.07	0.27	0.03	0.90	0.01	0.42	0.15	0.21	0.02	7e-6	0.39	0.46	0.01	0.58		0.19
111	0.45	0.86	0.69	0.46	0.01	0.93	0.55	1.00	0.92	0.57	0.12	0.43	0.68	0.58	0.73	0.28	0.19	

Samaria ancient Hebrew script experiment

This experiment addressed ostraca unearthed at Samaria ([3]; see examples in Fig 1), the capital of the Israelite kingdom [2]. This assemblage, comprised of ca. 100 short administrative texts, most probably dates to the first half of 8th century BCE [5–8]. They record the delivery of wine and oil from villages or royal estates in the countryside around Samaria to the capital. The original negatives of the inscriptions are located at the Harvard Semitic Museum.

The texts under examination are the 39 ostraca with low lines curvature: 2, 5, 6, 7, 8, 11, 12, 14, 15, 16a, 17a, 17b, 18, 19, 20, 21, 22, 24a, 29, 33, 34, 35, 36, 38, 40, 42, 43, 44, 45, 51, 52, 53, 54, 55, 56, 57, 59, 61 and 62. Among these, the relatively short ostraca 11, 15, 17b, 33, 34, 40, 44 and 61 were only used for statistical enrichment purposes (at the "Feature Extraction and Distance

Calculation" stage of the algorithm). All the other 31 inscriptions were utilized in the writers' differentiation examination. Various characteristics mentioned in the texts (i.e., the regnal year of the king, location, name of clan, commodity type, or mentioned personal name) can be seen in Table D; see also Fig 1.

Table D. Characteristics and reconstructed letter statistics of sampled Samaria texts. The shaded texts were reconstructed for features' statistics enrichment purposes only.

							Reconstructed letters							
**Ostracon No.**	**Field Registration No.**	**Year**	**Location**	**Clan**	**Addressee**	**Commodity**	***Bet***	***Yod***	***Lamed***	***Mem***	***Nun***	***Resh***	***Shin***	***Taw***
**2**	4583	10	Azzah		Gaddiyau		2		1			1	3	1
**5**	3863	9	Kozoh		Gaddiyau	Wine	1		2		1		1	1
**6**	3997	9	Kozoh		Gaddiyau	Wine		3	1		3		2	2
**7**	4578	9			Gaddiyau		1		1		1		2	1
**8**	3957	9	Geb[a]?		Ahino'am		2		1	2	2		1	1
**11**	4526				Ahino'am	Wine				1	1			
**12**	4525	9	Siphtan		Ba'alzamar	Wine	3	2	3		2	1	2	3
**14**	4608	9	Az[…]t Par'an		Shemaryau	Wine	2	2	2	1	3	2	4	2
**15**	4607		Hazeroth				1	1	1					1
**16a**	3891	10	Sepher		Gaddiyau	Oil	1	1	2	2	2	2	2	2
**17a**	3894	10	Azzah		Gaddiyau	Oil	2	1	2	1	2	2	3	2
**17b**	3899		Azzah		Gaddiyau	Oil			1		1	1	1	1
**18**	3931	10	Hazeroth		Gaddiyau	Oil	2		1	2	2	2	3	3
**19**	4031	10	Yazith		Ahino'am	Oil	1	1	2	1	1		2	2
**20**	3995	10	Kerm ha-Tell			Oil				2		2		
**21**	3889	10	Kerm ha-Tell?		Shemaryau	Oil	2	1	3	3	2	3	4	4
**22**	3932	15	Hazeroth	Helek	Asa son of Ahimelek		1		4	2			1	1
**24a**	3865, 3866	15	Hazeroth	Helek	Asa son of Ahimelek		1			1	1	1	2	1
**29**	4555, 4556, 4579	15	Sepher	Shemida	Asa son of Ahimelek							2		
**33**	3909	15		Shemida	Helez son of Gaddiyau					1				1
**34**	3903, 3933	15		Shemida	Helez son of Gaddiyau			1						1
**35**	39, 133, 914	15		Shemida	Helez son of Gaddiyau				2				2	
**36**	3902, 3906			Shemida	Ahima?	Wine		2		1				
**38**	3993	15		Shemida	Ahima				2	1			1	
**40**	4527	?		Shemida	A…			1	1	1				
**42**	3994	15		Asriel	Yeda'yau							2		
**43**	3875	15			Hanan Ba'ara		1		1		2			
**44**	3867	15	Shechem			Wine		1		1		1		
**45**	3896	15	Yazith	Hoglah	Hanan Ba'ara		2	2	1	1	3			1
**51**	4661	10						1					2	
**52**	4629	15					2							
**53**	3890	10	Kerm ha-Tell				2	1	2		3	2	3	3
**54**	4171	10	Kerm ha-Tell				2		2	1			2	2
**55**	4660	10				Oil	1	1	2	1		3	2	2
**56**	4617	15	Kerm ha-Tell?		Nimshi		1			1	1		2	1
**57**	4582			Shemida			1	2		1	1		1	
**59**	4581					Oil	2		1	1	2		1	1
**61**	3864	15					1		1	1		1	1	1
**62**	3934			Shemida		Wine		2		1	1		1	

All available letters with sufficient statistics were utilized: *bet, yod, lamed, mem, nun, resh, shin, taw*. The other letters did not produce sufficient instances for comparison purposes (as stated in sub-section C, we require at least "2 characters vs. 3 characters" comparison condition to be fulfilled; the theoretically possible "1 vs. 6" comparison condition was not met even once in the Samaria corpus). In total, 293 legible characters were restored, based on computerized images of the inscriptions. Statistics for the resulting letters for each text are summarized in Table D; all reconstructions are available in [17].

The complete results of the Samaria corpus analysis can be seen in in Table 1 of the main article. In addition, Table E provides contrasting statistics of Arad and Samaria experiments. It can be seen that most of the Samaria ostraca pairs did not have sufficient data for comparison. This occurred due to the brevity of the Samaria inscriptions, which contained a very small number of legible restored characters for each text (9.5 characters on average, compared to 23.7 in Arad), often not possessing adequate statistics for even a single letter comparison to take place. Nevertheless, 10 separations out of 138 comparisons were achieved (7.2% of separations out of all the comparisons). The number of separations in Samaria is much lower than in Arad (44 separations; i.e., 29.5% of the comparisons).

Table E. Statistics of Arad and Samaria writers' separation experiments.

Parameter	Arad	Samaria
Number of inscriptions	18	31
Number of restored characters	427	293
Average number of restored characters for each inscription	23.7	9.5
Number of pair-wise comparisons	149	138
Number of separations	44	10
% of separations	29.5%	7.2%
Minimal p-value	$2x10^{-6}$	0.03

There may be diverse reasons for the low number of separations in Samaria. First, the scarcity of data in Samaria may prohibit our writers' separation algorithm from achieving significant

conclusions. This may also be reflected in the fact that Samaria did not yield any *P* below 0.03, while in Arad, *P* as low as 2×10^{-6} were obtained. Second, the number of writers in Samaria may have been much lower than in Arad. In such a case, we would not expect many separations to be obtained. It is even possible that a single scribe in Samaria produced all the evaluated inscriptions, and *all* the identified separations are in fact False Positives.

In order to further investigate the separations obtained in Samaria, a more sophisticated evaluation of the results is required. Therefore, we propose an entirely new method to compute the most likely number of writers in Samaria via a Maximum Likelihood Estimation procedure. The details of this approach are provided in the next section.

Section 2: Estimating the Most Likely Number of Authors

Introduction

The end product of the writers' differentiation algorithm (described in detail in Section 1) is a table containing the *P* (p-values) for a comparison of each pair of ostraca. Using a pre-defined threshold of $P \leq 0.1$, we decide whether two given inscriptions represent distinct authorship. Such a result might be a realization of a true state, i.e., that the inscriptions were originally written by different writers (TP). However, some of these “separations” may come from False Positive detections of the algorithm (FP). As can be seen in Table B, empirically the FP scenario occurs with probability of *much less* than the expected 0.1. Nevertheless, false separations are possible, and might even be prominent in the results, e.g., if the number of writers is small. As demonstrated in Table 1 of the main text, the small number of writer differentiations achieved by our technique (10 out of 138 comparisons), indeed occurred within the *Inspected Corpus* of Samaria. The limited number of separations in Samaria, compared to Arad (7.2% vs. 29.5% of separations out of all the comparisons, accordingly), may have different explanations. Among them are the scarcity of data

in Samaria (on average, 9.5 restored characters per each inscription, compared to 23.7 in Arad), leading to less significant results. Additionally, the number of writers in Samaria may in fact be low – possibly as low as a single author. In fact, potentially *all* the separations represent False Positive outcomes of our algorithm. Conversely, if certain separations are true, the question is, *What is the most likely number of scribes in Samaria*?

The last inquiry leads us to develop a new Maximum Likelihood Estimation (MLE) framework, yielding the most likely number of scribes. The steps of the new scheme are as follows:

A. **Estimating True Negative (TN) and False Positive (FP)** via "same writer" simulations.

B. **Estimating True Positive (TP) and False Negative (FN**) via "different writer" simulations.

C. **Estimating the most likely number of writers** for the Inspected Corpus.

Steps A and B, estimating the *confusion matrices* (i.e., TN, FP, TP and FN) for a given corpus (in our case Samaria), cannot be performed on the corpus itself. Instead, they require an independent set of documents, denoted herein as *Estimation Corpus*. This corpus should be as similar as possible to the Inspected Corpus, which can be achieved by using texts that stem from approximately the same period, and entails identical medium, language and type of writing. Furthermore, these inscriptions should be accompanied by pre-established differentiations between their authors.

In Step C, the empirical distributions for “different number of writers” scenarios are estimated. E.g., assuming all the inscriptions were created by a single scribe, we conduct a Monte Carlo simulation in order to estimate its conditional probability density function (PDF), i.e. the probabilities of obtaining a total of 0 separations, 1 separation, 2 separations, etc. Assuming two writers in the corpus, we estimate another PDF. On the whole, we estimate the PDF's for scenarios ranging from a single writer to a number of writers equaling the number of inscriptions. A scenario maximizing the PDF value at the observed number of writers provides us with the ML estimate (MLE).

Moreover, a confidence level of $1-\alpha=0.95$ potentially provides all possible estimates for the number of writers.

Assumptions

Herein are the main assumptions laid in order to conduct the experiment with the described algorithm.

Assumption 1: **The corpus of Arad is suitable as the Estimation Corpus for the Inspected Corpus of Samaria**. Indeed, the Arad inscriptions are the richest among the Iron Age Hebrew inscriptions corpora, supplying a wealth of statistics; they are written in the same language upon the same medium (clay), utilizing the same scribal practice, i.e., the texts are written in ink and not incised. Although the two corpora are separated by ca. a century and a half, their scripts are quite similar (unlike the later scripts, practiced during the Second Temple period). In fact, although this is less crucial for our algorithm, even the contexts of these corpora, mainly dealing with daily logistical operation and a supply of commodities, are close parallels of each other.

Assumption 2: **Similarity of *P* statistics across different letters**. Within the writers' differentiation experiments (Section 1), the Arad and the Samaria corpora have different composition of reconstructed letters, serving as an input for the algorithm. In particular, both corpora include the reconstructed letters *yod*, *lamed*, *shin* and *taw*. Additionally, Arad has the letters *alep*, *he* and *waw*, while Samaria incorporates the letters *bet*, *mem*, *nun*, and *resh*. The assumption is that as a whole, the behavior of P stemming from different letters of Arad (the Estimation Corpus) is roughly similar. The mean P for all the letters is 0.448, with a standard deviation of 0.068; see Table F for additional information.

Table F. Statistics of *P* stemming from different Arad letters

	Alep	He	Waw	Yod	Lamed	Shin	Taw	Mean	Std
**#chars**	62	64	59	74	66	50	52	61	8.266
**AVG p-value**	0.376	0.470	0.508	0.458	0.346	0.440	0.539	**0.448**	**0.068**
**STD p-value**	0.289	0.321	0.338	0.326	0.325	0.295	0.318	**0.316**	**0.018**

Assumption 3: As in [1], we assume that each inscription of Arad was written by a single scribe. The same assumption is also made for the Samaria corpus.

Description of the Algorithm

The reconstructions of Samaria's characters (the Inspected Corpus' characters) allowed us to conduct 138 pair-wise comparisons of ostraca (see details in Section 1). These comparisons were based upon different compositions and quantities of letters. It is expected that the error rates (FP, FN) of the writers' differentiation algorithm would be dependent on the number of letter instances, as well as the number of letter types (as can be seen below, this expectation is confirmed in Table G). Thus, the confusion matrices need to be evaluated for each configuration of letter quantities existing in the Inspected Corpus separately. Following Assumption 2, we can construct a histogram of all possible unique configurations of the Inspected Corpus' comparisons, disregarding the letter types involved. Such a histogram is presented in Table G. For instance, the comparison configuration marked by "(2, 4)" occurs 21 times, and represents a comparison based upon a single letter type (e.g., *lamed*), with the first inscription having 2 instances of such letter, and the second inscription possessing 4 instances of the same letter. Furthermore, the configuration "(2, 3); (4, 2)," occurring once, represents a comparison of 2 vs. 3 characters of the same letter type, and 4 vs. 2 characters of another type. Note the order of the number of characters is important; e.g., the configuration "(2, 3); (2, 4)", is different from "(2, 3); (4, 2)." Indeed, comparing inscription A comprising 2 *lamed* and 2 *shin* with inscription B possessing 3 *lamed* and 4 *shin* is not the same as comparing inscription A comprising 2 *lamed* and 4 *shin* with inscription B possessing 3 *lamed* and

2 *shin*. On the other hand, for symmetry reasons, there is no need to count the occurrence of configurations "(2, 3); (4, 2)" and "(3, 2); (2, 4)" separately.

A. Estimating TN and FP

This step deals with the case of a "same writer" for the two tested inscriptions, which is the null-hypothesis (H_0) of the writers' differentiation algorithm (Section 1). If this is indeed the case, two outcomes of our algorithm are possible: either the algorithm mistakenly rejects H_0 (FP result); or it does not reject H_0 (TN result). Estimating the probabilities of FP and TN requires a set of document pairs, where *each pair is written by a single author*. Such a set is constructed by splitting sufficiently large inscriptions from the Estimation Corpus into two. Explicitly, for each letter configuration, we perform Monte Carlo simulations by sampling a suitable inscription from the Estimation Corpus (in our case the Arad corpus) and splitting it according to this configuration. Then, we use the writers' differentiation algorithm to record whether it provides an accurate outcome. These results are utilized in order to estimate FP and TN rates. The algorithm operates as follows:

```
ALGORITHM A
input Inspected_Configs, Estimation_Docs
MC_ITER = 1000
PVAL_THR = 0.1
FP_TN_confusion = {}
for cᵢ in Inspected_Configs:
       Suitable_Docs = Filter_Docs(cᵢ, Estimation_Docs)
       FP_count = 0; TN_count = 0
       for Monte_Carlo_Iter = 1 to MC_ITER:
              Random_Doc = Rand_Doc_Select(Suitable_Docs)
              Doc1, Doc2 = Rand_Docs_Construct(cᵢ, Random_Doc)
              pval       = Compare_Docs(Doc1, Doc2)
              if (pval<=PVAL_THR):
                     FP_count += 1
              else:
                     TN_count += 1
       FP_TN_confusion[cᵢ] = (FP_count/MC_ITER, TN_count/MC_ITER)
return FP_TN_confusion
```

Algorithm's details:

`Inspected_Configs` - the configurations within the Inspected Corpus; see Table G.

`Estimation_Docs` - the inscriptions of the Estimation Corpus, along with all the relevant data (e.g., letter quantities and characters' descriptors).

`MC_ITER` - number of Monte Carlo (MC) simulations, in our case 1000.

`PVAL_THR` - a pre-determined *P* threshold, in our case 0.1.

`FP_TN_confusion` - a placeholder for FP and TN estimated values for each configuration c_i.

`Filter_Docs(`c_i`, Estimation_Docs)` - a function filtering the `Estimation_Docs` from the Estimation Corpus, such that the amount of letters in each filtered document is sufficient to contain a given configuration c_i. E.g., for c_i="(2,5)", and a document with 8 instances of *lamed*, the matching is possible (since 2+5=7 characters of the same type are required), while in the case of a document with 6 instances of *shin* and 4 instances of *taw*, the matching is impossible.

`Rand_Doc_Select(Suitable_Docs)` - a function choosing at random a single document out of `Suitable_Docs`.

`Rand_Docs_Construct(`c_i`, Random_Doc)` - a function sampling letters and their instances from `Random_Doc`, and constructing two artificial documents according to the provided configuration c_i. Firstly, letters from c_i are randomly paired with letters of `Random_Doc`, according to their prominence ordering. E.g., for c_i="(2, 3); (2, 4)" and a `Random_Doc` with 9 *shin*, 8 *taw* and 5 *lamed*, at the beginning, the 2+4=6 characters will be randomly paired with either *shin* or *taw*; suppose *taw* is chosen. Then, the remaining 2+3=5 characters will be randomly paired with either *shin* or *lamed*; suppose *shin* is chosen. Secondly, artificial documents `Doc1` and `Doc2` are created by sampling (without returns) the paired letters (in fact, their descriptors, see Supplementary Material, Sub-section 1B) from `Random_Doc`, according to the c_i quantities. That is, in the previous example, 2 and 4 characters are sampled from *taw* instances, into `Doc1` and `Doc2`, respectively. Additionally, 2 and 3 characters are sampled into `Doc1` and `Doc2` from *shin* instances.

`Compare_Docs(Doc1, Doc2)` - a function calculating *P* for our null-hypothesis; see Supplementary Material, Sub-section 1C, for further details.

B. Estimating TP and FN

This step deals with the case of "different writers" for the two tested inscriptions. Again, H_0 assumes that the two given inscriptions were written by the same author. If this is indeed the case, two outcomes of our algorithm are possible: either the algorithm correctly rejects H_0 (TP result); or it does not reject H_0 (FN result). Estimating the probabilities of TP and FN requires a set of document pairs, where *the documents of each pair are known to be written by two different authors*. Explicitly, for each letter configuration, we perform Monte Carlo simulations through sampling two inscriptions pertaining to different scribes ("separated documents") from the Estimation Corpus. Then, we use the writers' differentiation algorithm to see whether it provides an accurate outcome. These results are utilized in order to estimate FN and TP rates. The algorithm operates as follows:

```
ALGORITHM B
input Inspected_Configs, Estimation_Docs
MC_ITER = 1000
PVAL_THR = 0.1
TP_FN_confusion = {}
for ci in Inspected_Configs:
       Suitable_Pairs = Filter_Pairs(ci, Estimation_Docs)
       TP_count = 0; FN_count = 0
       for Monte_Carlo_Iter = 1 to MC_ITER:
              Random_Pair = Rand_Pair_Select(Suitable_Pairs)
              Doc1, Doc2  = Rand_Docs_Construct(ci, Random_Pair)
              pval        = Compare_Docs(Doc1, Doc2)
              if (pval<=PVAL_THR):
                     TP_count += 1
              else:
                     FN_count += 1
       TP_FN_confusion[ci] = (TP_count/MC_ITER, FN_count/MC_ITER)
return TP_FN_confusion
```

Algorithm's details:

Inspected_Configs - the configurations within the Inspected Corpus; see Table G.

Estimation_Docs - the inscriptions of the Estimation Corpus, along with all the relevant data (e.g., letter quantities, characters' descriptors, as well as the presumed separations).

MC_ITER - number of Monte Carlo simulations, in our case 1000.

PVAL_THR - a pre-determined p-value threshold, in our case 0.1.

TP_FN_confusion - a placeholder for TP and FN estimated values for each configuration c_i.

Filter_Pairs(c_i, Estimation_Docs) - a function filtering pairs of documents *written by different authors* from the Estimation Corpus, such that the number of letters in each pair of filtered documents is sufficient to contain a given configuration c_i. E.g., for c_i="(2,5)", and a pair of "separated" documents, inscription A with 3 instances and inscription B with 9 instances of *lamed*, it is possible to match 2 to A (since 2<3), and 5 to B (since 5<9) - but not vice versa.

Rand_Pair_Select(Suitable_Pairs) - a function choosing at random a pair of documents out of Suitable_Pairs.

Rand_Docs_Construct(c_i, Random_Pair) - a function sampling letters and their instances from Random_Pair and constructing two artificial documents according to the provided configuration c_i. Firstly, letters from c_i are randomly paired with letters of Random_Pair. E.g., assume c_i="(2, 3); (2, 4)" and a Random_Pair containing inscription A with 4 *lamed* and 5 *shin*, and inscription B containing 4 *lamed* and 4 *shin*. In such a case, assigning *lamed* to (2,3) and *shin* to (2,4), as well as assigning *lamed* to (2,4) and *shin* to (2,3), would be considered (since the letter statistics are sufficient in both cases); suppose the second option is randomly chosen. Secondly, artificial documents Doc1 and Doc2 are created by sampling (without returns) the paired letters (in fact, their descriptors, see Supplementary Material, Sub-section 1B) from Random_Pair, according to the c_i quantities. That is, in the previous example, 2 *shin* and 2 *lamed* would be sampled from inscription A into Doc1, while 3 *shin* and 4 *lamed* would be sampled from inscription B into Doc2.

`Compare_Docs(Doc1, Doc2)` - a function calculating P for our null-hypothesis; see Supplementary Material, Sub-section 1C, for further details.

C. Estimating the most likely number of writers within the Inspected Corpus

A combination of the outcomes of steps A and B results in **confusion matrices** for each configuration of the Inspected Corpus (in our case, Samaria). Based upon these matrices, we can *estimate the most likely number of writers within the Inspected Corpus*. In order to provide an MLE, we need to estimate the conditional probabilities for the number of separations achieved (S), assuming varying number of writers W - i.e., $\Pr(S \mid W)$. We estimate $\Pr(S \mid W)$ using Monte Carlo (MC) simulations. Explicitly, for $W = n$, a single MC iteration comprises a random attribution of the inscriptions (of the inspected corpus) to n simulated scribes; thereafter, for each pair of inscriptions, we draw a separation according to the probabilities of the corresponding confusion matrix. For example, given a comparison between ostraca A and B originating from 2 different simulated scribes and having a single shared letter type with 3 vs. 4 instances, we utilize the corresponding TP and FN rates (see Table G, "main simulation" results) and record whether a separation occurs (in that case the probability for a separation to occur is 36%). The algorithm operates as follows:

```
ALGORITHM C
input Confusion_Matrices, Inspected_Docs, Obs_N_Seps
MC_ITER = 100000
CONF    = 0.1
Seps_Hist = {}
for N_Writers = 1 to length(Inspected_Docs):
       for Monte_Carlo_Iter = 1 to MC_ITER:
              Docs_Wr = Rand_Writers_Assign(Inspected_Docs, N_Writers)
              N_Seps  = Rand_Seps(Wr_Docs, Confusion_Matrices)
              Seps_Hist[N_Seps,N_Writers] += 1
MLE_N_Writers, Conf_Intervs = MLE(Seps_Hist, Obs_N_Seps, CONF)
return MLE_N_Writers, Conf_Intervs
```

Algorithm's details:

`Confusion_Matrices` - joined results of steps A and B.

`Inspected_Docs` - the inscriptions of the Inspected Corpus, along with all the relevant data (e.g., letter quantities).

`Obs_N_Seps` - the observed number of separations in the Inspected Corpus.

`MC_ITER` - number of Monte Carlo (MC) simulations, in this case 100,000.

`CONF` - a required two-sided confidence value; in our case 0.1 (which results in at most 0.05 allowed to be "cropped" from each side of the histogram)

`Seps_Hist` - a placeholder (matrix) counting the occurrences of results in MC simulations, with the rows corresponding to the simulated "number of writers" and the columns corresponding to the "number of separations" obtained during the simulations. E.g., if a scenario of `N_Writers=3` yielded 9 separations (`N_Seps=9`) within 15 different MC simulations (out of `MC_ITER`), we get `Seps_Hist[9,3]=15`; if the same scenario (`N_Writers=3`) yielded a single separation twice, we get `Seps_Hist[1,3]=2`, etc.

`Rand_Writers_Assign(Inspected_Docs, N_Writers)` - a function randomly assigning `Inspected_Docs` to `N_Writers`; at least one document is assigned to each writer. The output is `Docs_Wr`, an assignment of documents into writers.

`Rand_Seps(Docs_Wr, Confusion_Matrices)` - a function inspecting the `Docs_Wr`, and drawing separations according to "same writer" or "different writers" scenarios for each pair of documents, taking into account the FP vs. TN, and TP vs. FN probabilities for the appropriate pair's configuration in the `Confusion_Matrices`. The output is the total number of separations, `N_Seps`.

`MLE(Seps_Hist, Obs_N_Seps, CONF)` - a function deriving the Maximum Likelihood Estimate, provided the observed number of separations within the Inspected Corpus, `Obs_N_Seps`. The function chooses `N_Writers` such that `Seps_Hist[Obs_N_Seps, N_Writers]` is maximal for the

given `Obs_N_Seps`. Additionally, for each "number of writers", appropriate confidence intervals are calculated from the `Seps_Hist`, according to `CONF`.

Experimental details and results

We applied the algorithm on the Inspected Corpus of Samaria and the Estimation Corpus of Arad. The Estimation Corpus was fed to Step B of the algorithm, under the assumption that the writers' differentiations obtained for Arad in Section 1 are valid. In other words, we neglect the FP and FN occurrences within these results. The outcomes of Steps A and B are provided in Table G ("main simulation"). For example, taking the Arad separations, in case we compare a single letter type with 3 vs. 2 instances, the FP rate is 2.2% and the FN rate is 78.6%. Whereas, upon comparing two letter types, one with 2 vs. 3 instances and the other with 3 vs. 3 instances, the algorithm yielded FP rate of 0.7% and FN rate of 74.3%. As can be seen in Table G, the low rates of FP and high rates of FN are consistent throughout all letter configurations. In other words, the writers' differentiation algorithm is careful in identifying distinct authorship, but, if it does indicate authors' dissimilarity, it is very likely to be true.

The results of Step C are shown in Fig 4 and provided in Dataset S2. It appears that **the ML estimation of the number of writers in Samaria is 2**. Within a symmetrical confidence interval of 95% (i.e., removing 2.5% of the samples from each side of the histogram), the only valid simulation is 2 writers.

Although we consider this result as trustworthy, it is possible that the writers' differentiation statistics, obtained for Arad in Section 1 somehow misrepresents the status of separations within the Arad corpus at 600 BCE. This may happen, for example, due to different writers that yield insignificant results in the writers' differentiation algorithm, and thus produce a false misdetection (FN). This, in turn, may skew the MLE procedure. Thus, an **alternative simulation** aiming at

estimating *an upper bound* on the number of authors was conducted, raising the *P* threshold **for the Estimation Corpus (Arad) only** to 0.2 instead of 0.1. The expected results are not easy to predict, since the alternative simulation may have introduced FN cases such as described above, but it may have also introduced TN cases, skewing the results in the opposite direction. The confusion matrices for the main and the alternative simulations are provided in Table G. All the results pertaining to ML calculation are also provided in Dataset S2 and shown in Fig 4 (main simulation) and Fig A (alternative simulation). **The ML estimation of two writers in Samaria is maintained in the alternative simulation**. Moreover, within a symmetrical confidence interval of 95% (i.e., removing 2.5% of the samples from each side of the histogram), the only valid simulation is again 2 writers. Hence, we conclude that the results obtained in the "main" simulation are sound.

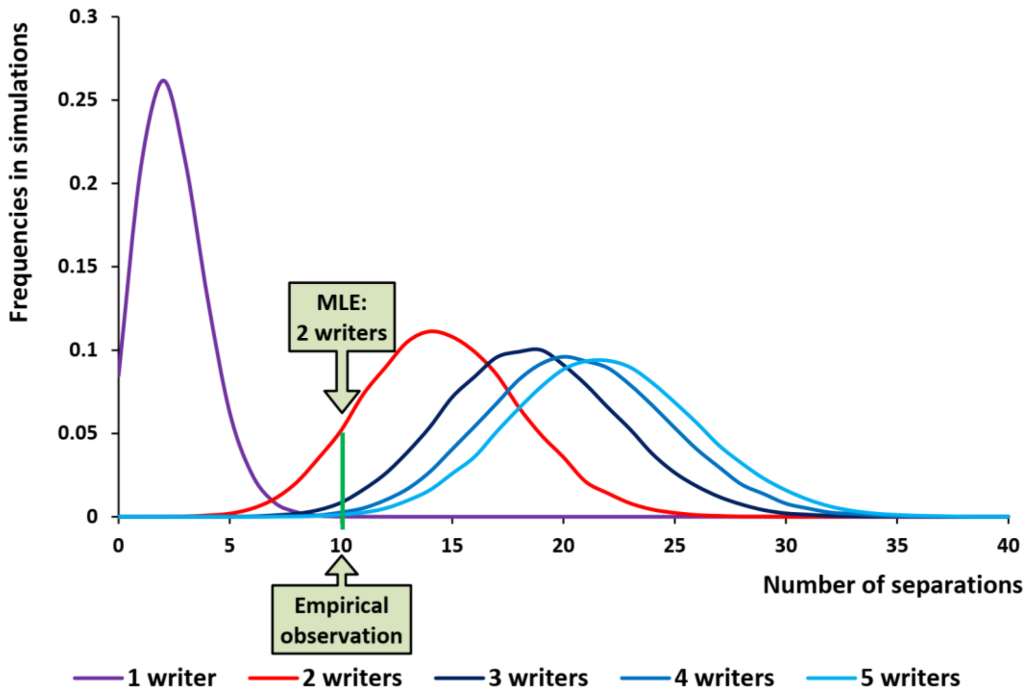

Fig A. The results of alternative simulation (cf. the almost identical Fig 4 for the main one). Conditional PDF for the number of separations in our ostraca sample, with different simulated number of writers. The MLE of two authors is maintained.

Table G. Samaria configurations, their counts and the resulting confusion matrices, for both the main and the alternative Monte Carlo simulations (see text for details)

Samaria Configuration	Count	FP%	TN%	Main simulation (Arad threshold=0.1)		Alternative simulation (Arad threshold=0.2)	
				TP%	FN%	TP%	FN%
(2, 3)	63	2.2	97.8	21.4	78.6	21.7	78.3
(2, 4)	21	2.1	97.9	18.8	81.2	16.2	83.8
(2, 3), (2, 3)	9	1.3	98.7	10.9	89.1	10.6	89.4
(3, 3)	5	1.2	98.8	25.6	74.4	22.3	77.7
(2, 3), (3, 2)	4	0.7	99.3	9	91	9	91
(2, 3), (2, 3), (3, 2)	4	0.4	99.6	9.9	90.1	11	89
(3, 4)	3	5.1	94.9	36	64	36.6	63.4
(2, 3), (2, 4)	2	2.8	97.2	18.7	81.3	16.8	83.2
(2, 3), (3, 3)	2	0.7	99.3	25.7	74.3	24.8	75.2
(2, 3), (2, 3), (2, 3)	2	0.9	99.1	10	90	9.6	90.4
(2, 3), (2, 3), (3, 3)	2	0.2	99.8	12	88	13.2	86.8
(2, 3), (2, 4), (2, 4)	2	1.4	98.6	17.1	82.9	17	83
(2, 3), (3, 4)	1	1.6	98.4	26.5	73.5	26.4	73.6
(2, 3), (4, 2)	1	1.2	98.8	19.8	80.2	18.1	81.9
(2, 3), (4, 3)	1	1.5	98.5	26.7	73.3	30.1	69.9
(2, 3), (3, 2), (3, 3)	1	0.4	99.6	13.6	86.4	13.9	86.1
(2, 3), (3, 2), (4, 3)	1	1	99	24	76	27.5	72.5
(2, 3), (3, 3), (3, 3)	1	0.8	99.2	17.5	82.5	18.3	81.7
(2, 3), (3, 3), (4, 3)	1	0.9	99.1	32.1	67.9	28.7	71.3
(2, 4), (2, 4), (3, 2)	1	0.7	99.3	16.3	83.7	18.2	81.8
(2, 4), (3, 2), (3, 3)	1	0.4	99.6	15	85	14.7	85.3
(2, 3), (2, 3), (2, 3), (3, 2)	1	0.2	99.8	11	89	11.6	88.4
(2, 3), (2, 3), (2, 4), (3, 4)	1	1.1	98.9	24.9	75.1	23	77
(2, 3), (2, 3), (3, 4), (3, 4)	1	0.6	99.4	32.7	67.3	35.2	64.8
(2, 3), (2, 4), (2, 4), (3, 3)	1	0.6	99.4	17.7	82.3	17.2	82.8
(2, 4), (3, 2), (3, 3), (3, 4)	1	0.8	99.2	23.7	76.3	25.4	74.6
(2, 3), (2, 3), (2, 3), (2, 4), (2, 4)	1	0.5	99.5	15.4	84.6	12.1	87.9
(2, 3), (2, 3), (2, 4), (3, 2), (4, 4)	1	0.4	99.6	19.9	80.1	21.6	78.4
(2, 3), (2, 3), (3, 2), (3, 2), (3, 3)	1	0	100	12.2	87.8	13.5	86.5
(2, 3), (2, 4), (3, 2), (3, 2), (3, 2)	1	0	100	10.1	89.9	10.9	89.1
(2, 3), (3, 2), (3, 2), (4, 3), (4, 3)	1	0.9	99.1	28.1	71.9	26.5	73.5

References

1. Faigenbaum-Golovin S, Shaus A, Sober B, Levin D, Na'aman N, Sass B, et al. Algorithmic handwriting analysis of Judah's military correspondence sheds light on composition of biblical texts. Proc Natl Acad Sci USA. 2016; 113: 4664-4669. https://doi.org/10.1073/pnas.1522200113
2. Finkelstein I. The Forgotten Kingdom. The Archaeology and History of Northern Israel. Atlanta: Society of Biblical Literature. 2013.
3. Reisner GA, Fischer CS, Lyon DG. Harvard Excavations at Samaria. Cambridge, MA: Harvard University Press. 1924.
4. Aḥituv S. Echoes from the Past: Hebrew and Cognate Inscriptions from the Biblical Period. Jerusalem: Carta. 2008.
5. Aharoni Y. The Land of the Bible: A Historical Geography. Louisville, KY: Westminster John Knox Press. 1979.
6. Lemaire A. Les ostraca hébreux de l'époque royale Israélite. Paris, 1973.
7. Lemaire A. Inscriptions hébraques, Vol. 1: Les ostraca. Littératures anciennes du Proche-Orient 9. Paris: Cerf. 1977.
8. Rainey AF. Toward a precise date for the Samaria ostraca. Bull Am Schools Orient Res. 1988; 272: 69-74.
9. Tappy RE. The Archaeology of the Ostraca House at Israelite Samaria: Epigraphic Discoveries in Complicated Contexts. ASOR 70. Boston: American Schools of Oriental Research. 2016.
10. USGS EROS (Earth Resources Observatory and Science (EROS) Center) (public domain): https://www.usgs.gov/centers/eros
11. Yadin Y. Recipients or owners? A note on the Samaria ostraca. Isr Explor J. 1959; 9: 184–187.
12. Rendsburg G. Diglossia in Ancient Hebrew. American Oriental Series 72. New Haven, CT: American Oriental Society. 1990.
13. Kaufman IT. Samaria ostraca. In Freedman DN, editor. The Anchor Bible Dictionary, Vol. 5. New York, NY: Doubleday. 1992. pp 921–926.
14. Niemann HM. A new look at the Samaria ostraca: The king-clan relationship. Tel Aviv. 2008; 35: 249–266.
15. Shaus A. Turkel E. Writer identification in modern and historical documents via binary pixel patterns, Kolmogorov-Smirnov test and Fisher's method. J Imaging Sci Technol. 2017; 61: 010404-1–010404-9. https://doi.org/10.2352/J.ImagingSci.Technol.2017.61.1.010404
16. Faigenbaum-Golovin S. Levin D. Piasetzky E. Finkelstein I. Writer characterization and identification of short modern and historical documents: reconsidering paleographic tables. Proceedings of the 19th ACM Symposium on Document Engineering (DocEng 2019), 23:1–23:4.
17. Samaria Ancient Hebrew dataset, figshare (CC BY 4.0 license): https://doi.org/10.6084/m9.figshare.10266206.v1
18. Faigenbaum S, Sober B, Shaus A, Moinester M, Piasetzky E, Bearman G, et al. Multispectral images of ostraca: Acquisition and analysis. J Archaeol Sci. 2012; 39: 3581-3590. https://doi.org/10.1016/j.jas.2012.06.013

19. Sober B, Faigenbaum S, Beit-Arieh I, Finkelstein I, Moinester M, Piasetzky E, et al. Multispectral imaging as a tool for enhancing the reading of ostraca. Palestine Exploration Quarterly. 2004; 146: 185-197. https://doi.org/10.1179/0031032814Z.000000000101
20. Faigenbaum-Golovin S, Mendel-Geberovich A, Shaus A, Sober B, Cordonsky M, Levin D, et al. Multispectral imaging reveals biblical-period inscription unnoticed for half a century. PLOS ONE. 2017; 12: e0178400. https://doi.org/10.1371/journal.pone.0178400
21. Mendel-Geberovich A, Shaus A, Faigenbaum-Golovin S, Sober B, Cordonsky M, Piasetzky E, et al. A brand new old inscription: Arad ostracon 16 rediscovered via multispectral imaging. Bull Am Schools Orient Res. 2017; 378: 113-125. https://doi.org/10.5615/bullamerschoorie.378.0113
22. Faigenbaum S, Sober B, Finkelstein I, Moinester M, Piasetzky E, Shaus A, et al. Multispectral imaging of two Hieratic inscriptions from Qubur el-Walaydah. Egypt and the Levant. 2014; 24: 349-353. https://doi.org/10.1553/s349
23. Faigenbaum S, Sober B, Moinester M, Piasetzky E, Bearman G. Multispectral imaging of Tel Malhata ostraca. In Beit-Arieh I, Freud L, editors. Tel Malhata: A Central City in the Biblical Negev. Tel Aviv: Tel Aviv University. 2015. pp. 510–513.
24. Shaus A, Sober B, Tzang O, Ioffe Z, Cheshnovsky O, Finkelstein I. Raman binary mapping of Iron Age ostracon in unknown material composition and high fluorescence setting – A proof of concept, Archaeometry. 2019; 61, 459–469. https://doi.org/10.1111/arcm.12419
25. Shaus A, Turkel E, Piasetzky E, Binarization of First Temple period inscriptions - Performance of existing algorithms and a new registration based scheme. Proceedings of the 13th International Conference on Frontiers in Handwriting Recognition (ICFHR 2012), 641–646. https://doi.org/10.1109/ICFHR.2012.187
26. Shaus A, Sober B, Turkel E, Piasetzky E. Improving binarization via sparse methods. Proceedings of the 16th International Graphonomics Society Conference (IGS 2013), 163–166.
27. Bar-Yosef I, Beckman I, Kedem K, Dinstein I. Binarization, character extraction, and writer identification of historical Hebrew calligraphy documents. Int J Doc Anal Recognit. 2007; 9: 89-99.
28. Bulacu M, Schomaker L. Text-independent writer identification and verification using textural and allographic features. IEEE Trans Pattern Anal Mach Intell. 2007; 29: 701-717.
29. Panagopoulos M, Papaodysseus C, Rousopoulos P, Dafi D. Tracy S. Automatic writer identification of ancient Greek inscriptions. IEEE Trans Pattern Anal Mach Intell. 2009; 31: 1404-1414.
30. Fecker D, Asit A, Märgner V, El-Sana J, Fingscheidt T, Writer identification for historical Arabic documents. Proceedings of the 22nd International Conference on Pattern Recognition (ICPR 2014), 3050-3055.
31. Dhali M, He S, Popovic M, Tigchelaar E, Schomaker L. A digital palaeographic approach towards writer identification in the Dead Sea scrolls. Proceedings of the 6th International Conference on Pattern Recognition Applications and Methods (ICPRAM 2017), 693-702.

32. Dahllöf M, Automatic scribe attribution for medieval manuscripts. Digital Medievalist. 2018; 11: 1-26.
33. Aharoni Y, Arad Inscriptions. Jerusalem: Israel Exploration Society. 1981.
34. Sober B, Levin D. Computer aided restoration of handwritten character strokes. Computer-Aided Design. 2017; 89: 12-24.
35. Mumford D, Shah J. Optimal approximations by piecewise smooth functions and associated variational problems. Commun Pure Appl Math. 1989; 42: 577–685.
36. Kass M, Witkin A, Terzopoulos D. Snakes: Active contour models. Int J Comput Vis. 1988; 1: 321–331.
37. Rollston CA. The script of Hebrew Ostraca of the Iron Age: 8th–6th centuries BCE, PhD thesis, Johns Hopkins University, Baltimore. 1999.
38. Lowe DG. Distinctive image features from scale-invariant keypoints. Int J Comput Vis. 2004; 60: 91–110.
39. Markus D, Sablatnig R. Recognition of degraded handwritten characters using local features. Proceedings of the 10th International Conference on Document Analysis and Recognition (ICDAR 2009), 221-225.
40. Tahmasbi A, Saki F, Shokouhi SB. Classification of benign and malignant masses based on Zernike moments. Comput Biol Med 41. 2011; 726–735.
41. Tahmasbi A. Zernike moments. 2012. Available at www.mathworks.com/matlabcentral/fileexchange/38900-zernike-moments.
42. Sexton A, Todman A, Woodward K. Font recognition using shape-based quadtree and kd-tree decomposition. Proceedings of the 3rd International Conference on Computer Vision, Pattern Recognition and Image Processing (CVPRIP 2000), 212–215.
43. Armon S. Descriptor for shapes and letters (feature extraction). 2012. Available at www.mathworks.com/matlabcentral/fileexchange/35038-descriptor-for-shapes-andletters-feature-extraction.
44. Trier ØD, Jain AK, Taxt T. Feature extraction methods for character recognition—A survey. Pattern Recognit. 1996; 29: 641–662.
45. Shaus A, Turkel E, Piasetzky E. Quality evaluation of facsimiles of Hebrew First Temple period inscriptions. Proceedings of the 10th IAPR International Workshop on Document Analysis Systems (DAS 2012), 170–174. https://doi.org/10.1109/DAS.2012.70
46. Shaus A, Faigenbaum-Golovin S, Sober B, Turkel E, Piasetzky E. Potential contrast - A new image quality measure, In Proceedings of the IS&T International Symposium on Electronic Imaging 2017, Image Quality and System Performance XIV Conference (IQSP 2017), 52-58. https://doi.org/10.2352/ISSN.2470-1173.2017.12.IQSP-226
47. Fisher RA. Statistical Methods for Research Workers. Edinburgh: Oliver and Boyd. 1925.
48. Goren Y, Finkelstein I, Na'aman N. Inscribed in Clay: Provenance Study of the Amarna Tablets and other Ancient Near Eastern Texts. Tel Aviv University Monograph Series 28. Tel Aviv: Tel Aviv Univ. 2004.
49. Meshel Z. Kuntillet ʻAjrud (Ḥorvat Teman): An Iron Age II Religious Site on the Judah-Sinai Border. Jerusalem: Israel Exploration Society. 2012.

50. Finkelstein I, Sass B. The West Semitic alphabetic inscriptions, Late Bronze II to Iron IIA: Archeological context, distribution and chronology. Hebrew Bible and Ancient Israel. 2013; 2: 149-220.
51. Sass B, Finkelstein I. The swan-song of Proto-Canaanite in the ninth century BCE in light of an alphabetic inscription from Megiddo. Semitica et Classica. 2016; 9: 19-42.
52. Mendel A. Epigraphic lists in Israel and its neighbors in the First Temple period, PhD thesis, Hebrew University, Jerusalem. 2014.
53. Mazar A. Excavations at Tel Beth-Shean 1989–1996: Vol. I: From the Late Bronze Age IIB to the Medieval Period. The Beth-Shean Valley Archaeological Project Publications 1. Jerusalem: Israel Exploration Society. 2006.
54. Torczyner H. Lachish I: The Lachish Letters. London and New York: Oxford University Press. 1938.
55. Beit-Arieh I. Horvat ‘Uza and Horvat Radum: Two Fortresses in the Biblical Negev. Tel Aviv University Monograph Series 25. Tel Aviv: Tel Aviv University. 2007.
56. Beit-Arieh I, Freud L. Tel Malhata: A Central City in the Biblical Negev. Tel Aviv University Monograph Series 32. Tel Aviv: Tel Aviv University. 2015.
57. Faigenbaum-Golovin S, Rollston CA, Piasetzky E, Sober B, Finkelstein I. The Ophel (Jerusalem) ostracon in light of new multispectral images. Semitica. 2015; 57: 113-137.
58. Naveh J. A Hebrew letter from the seventh century B.C. Isr Explor J. 1960; 10: 129–139.
59. Mendel-Geberovich A, Faigenbaum-Golovin S, Shaus A, Sober B, Cordonsky M, et al. A renewed reading of Hebrew ostraca from Cave A-2 at Ramat Beit Shemesh (Nahal Yarmut), based on multispectral imaging. Vetus Testamentum. 2019; 69: 682-701. https://doi.org/10.1163/15685330-00001370
